# Supplementary figures and images for: Integrating when and what information in the left parietal lobe allows language rule generalization
Source: PLoS Biol. 2020 Nov 2;18(11):e3000895. doi: 10.1371/journal.pbio.3000895 (PMC7660506; doi:10.1371/journal.pbio.3000895)

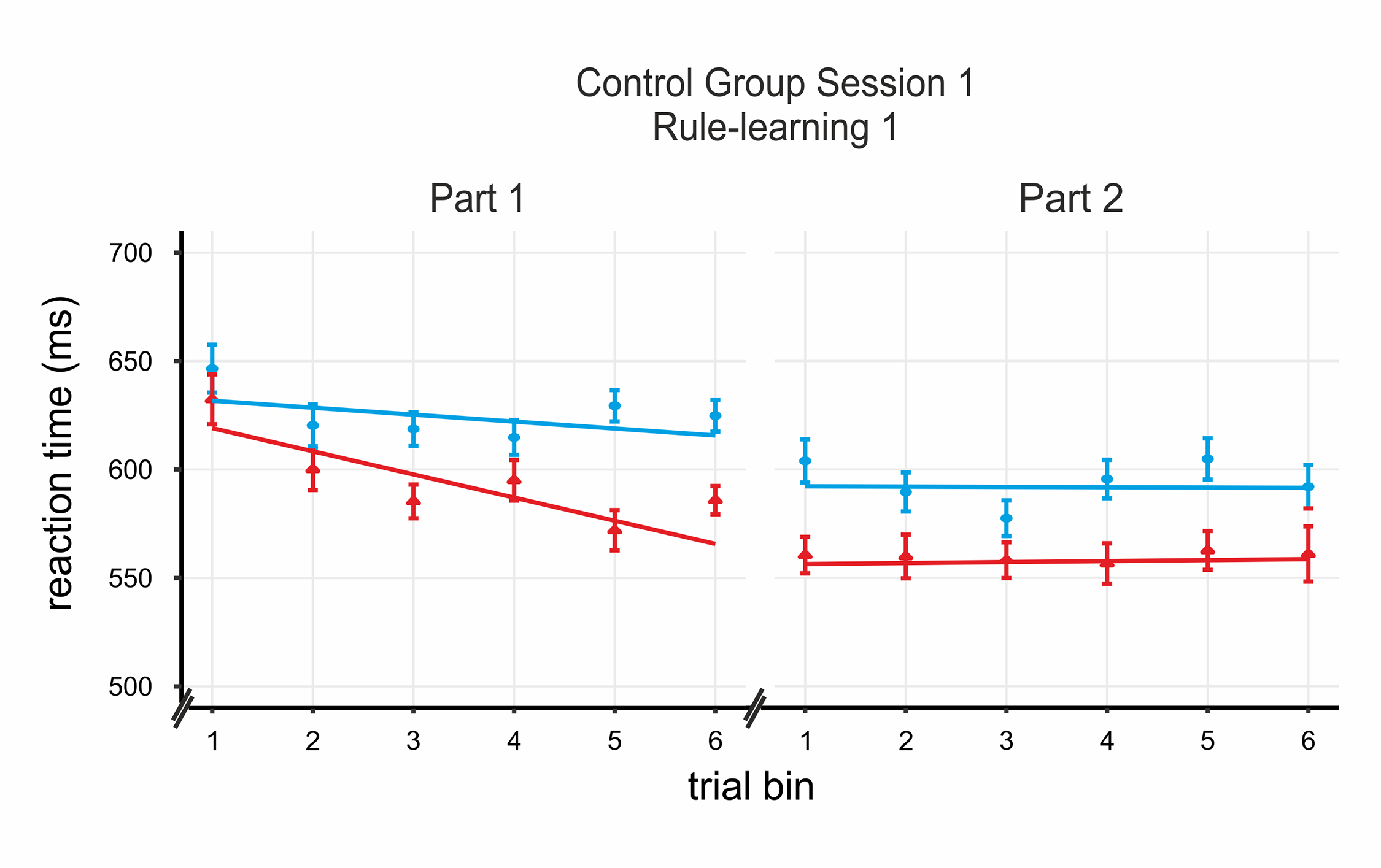

Supplement: S1 Fig — Slopes for rule and no-rule blocks over task repetitions derived from the mixed model analysis. The control group showed the expected transition from a significant learning slope in Part 1 (βdiff = −0.8, t = −3.1, p < 0.002) to a nonsignificant learning slope (βdiff = 0.06, t = 0.252, p > 0.8) with a significant rule effect in Part 2 (t[30] = 4.49, p < 0.001) and Part 1 (t[29] = 3.6, p < 0.002). Actual data shown averaged into 6 trial bins (for visual purposes only; the analysis did not bin the data) with the SEM over the slopes for rule and no rule derived from the mixed model analysis. Data used to generate S1 Fig can be found in S4 Data. SEM, standard error of the mean (TIF) [file pbio.3000895.s001.tif]

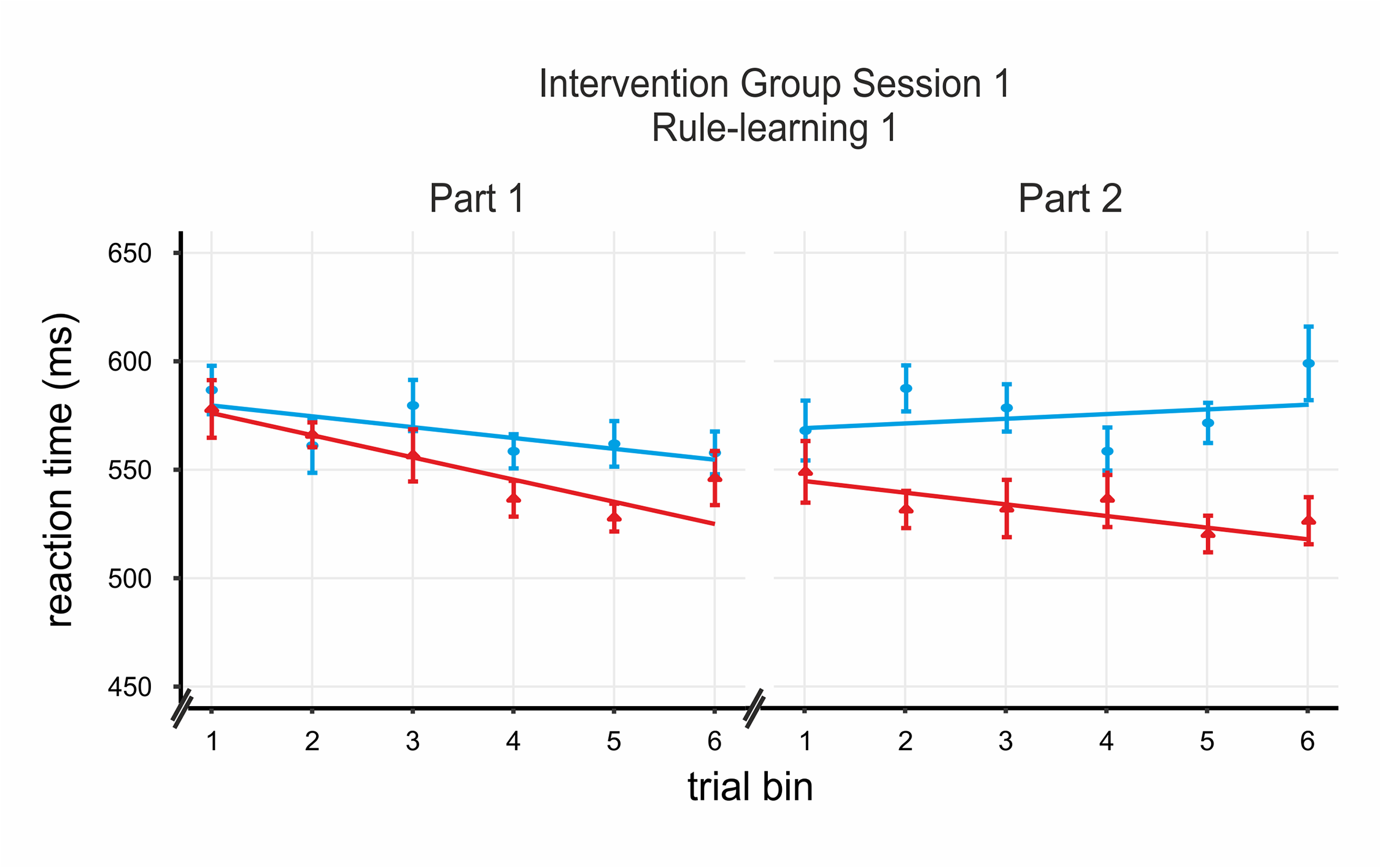

Supplement: S2 Fig — Slopes for rule and no-rule blocks over task repetitions derived from the mixed model analysis. The intervention group—perhaps owing to the smaller sample and/or scanner effects—appeared to comprise slower learners and still showed a significant learning slope during the fMRI phase (Part 2: βdiff = −0.8, t = −2.63, p < 0.01; Part 1: βdiff = −0.56, t = −2.13, p < 0.034), as well as the expected rule effects (Part 1: t[16] = 2.19, p < 0.044; Part 2: t[19] = 3.08, p < 0.007). Actual data shown averaged into 6 trial bins (for visual purposes only; the analysis did not bin the data) with the SEM over the slopes for rule and no rule derived from the mixed model analysis. Data used to generate S2 Fig can be found in S5 Data. fMRI, functional MRI; SEM, standard error of the mean (TIF) [file pbio.3000895.s002.tif]

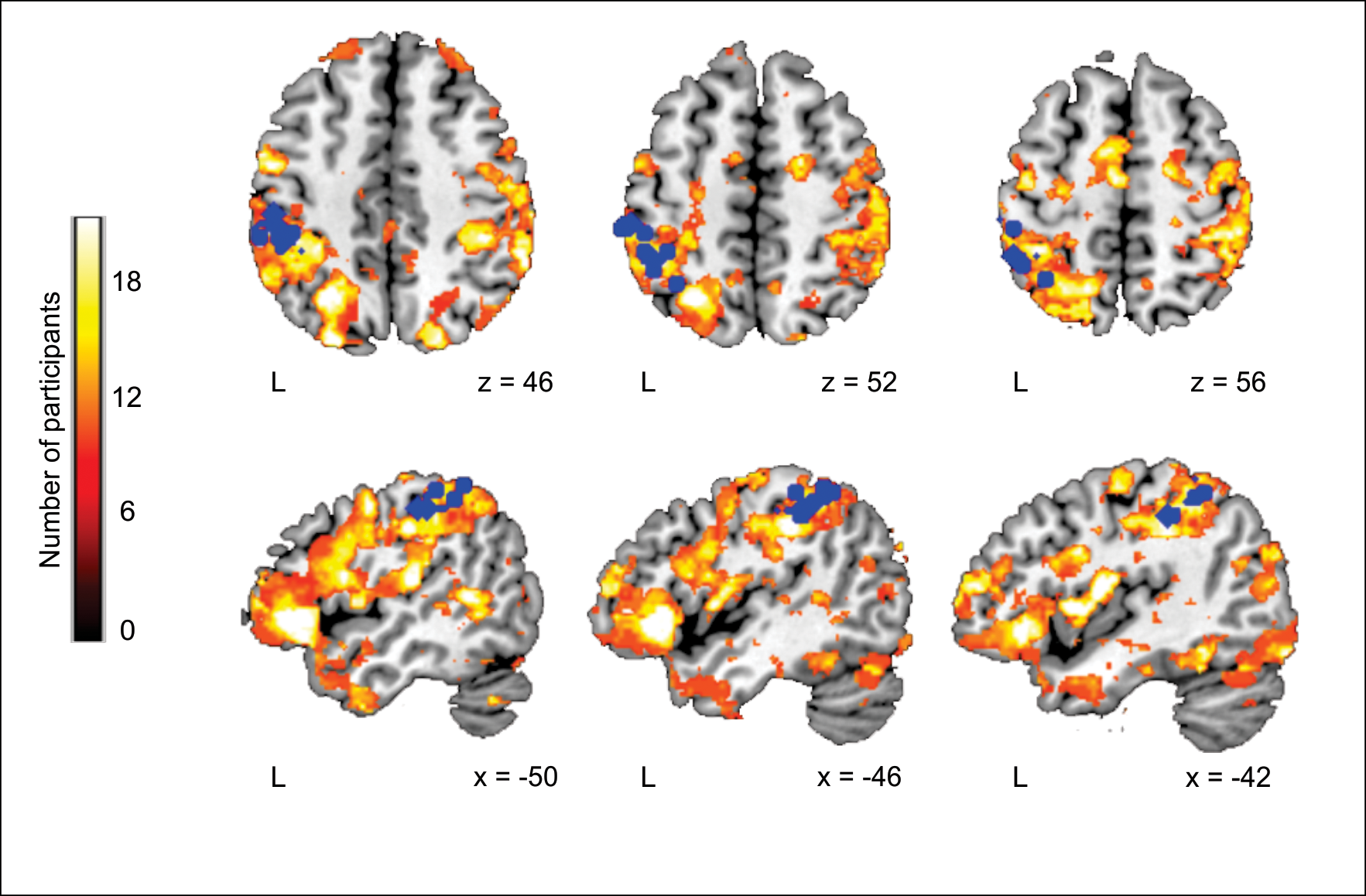

Supplement: S3 Fig — In red-yellow, overlap of individual masks for each participant's activation pattern. Only voxels in which at least 10 participants showed individual fMRI-enhanced activity during rule learning are shown. In blue, the sites for rTMS stimulation for all participants is shown (for clarity purposes, 4-mm spheres were created around the stimulation centers). Neurological convention is used, with MNI coordinates shown at the bottom right of each slice. fMRI, functional MRI; MNI, Montreal Neurological Institute; rTMS, repetitive transcranial magnetic stimulation (TIF) [file pbio.3000895.s003.tif]

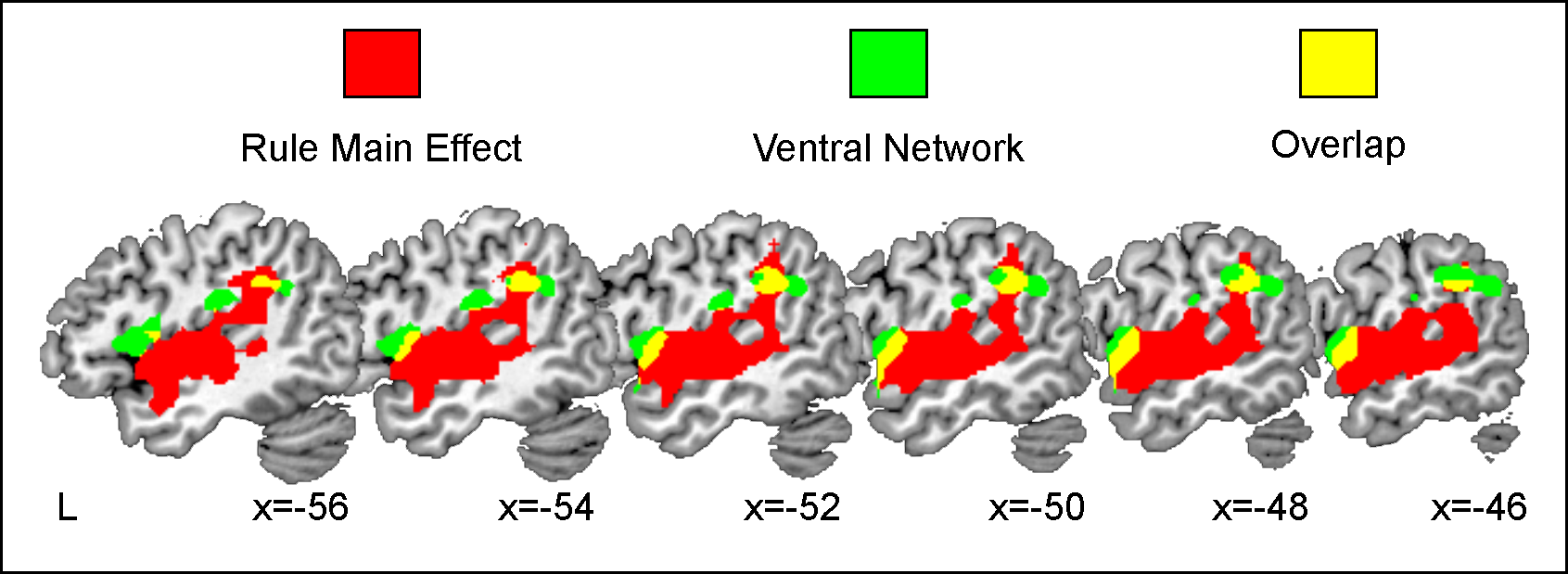

Supplement: S4 Fig — In the lPL, there is an overlap between the contrast showing regions in which the BOLD signal significantly covaries with the measure of statistical learning (learning slope) and the contrast showing the brain regions in which activity increases during rule blocks: the more activity in the lPL during rule blocks, the greater (that is, more negative) the slope, and the faster the statistical learning occurs. Only significant results (p < 0.05 FWE-corrected at the cluster level, with an additional p < 0.005 at the voxel level and 50 voxels of cluster extent) are shown over a canonical template with MNI coordinates on the bottom right of each slice. BOLD, blood-oxygenation-level–dependent; FWE, family-wise error; L, Left Hemisphere; lPL, left parietal lobe; MNI, Montreal Neurological Institute (TIF) [file pbio.3000895.s004.tif]
