# Supplementary material for: Integrating when and what information in the left parietal lobe allows language rule generalization
Source: PLoS Biol. 2020 Nov 2;18(11):e3000895. doi: 10.1371/journal.pbio.3000895 (PMC7660506; doi:10.1371/journal.pbio.3000895)
Supplement: S1 Table — Group-level fMRI local maxima for areas correlating with negative (that is, RTs are still decreasing) rule slopes during the rule blocks in the fMRI phase (see also red-yellow regions in Fig 4A, main text). Results are reported at a FWE p < 0.05 corrected threshold at the cluster level with 50 voxels of cluster extent, with an additional uncorrected p < 0.005 threshold at the voxel level. MNI coordinates were used. BA, Brodmann Area; fMRI, functional MRI; FWE, family-wise error; MNI, Montreal Neurological Institute; RT, reaction time (DOCX) [file pbio.3000895.s005.docx]

**S1 Table. Whole brain fMRI activity related to individual differences in rule slopes.** Group-level fMRI local maxima for areas correlating with negative (i.e. RTs are still decreasing) rule slopes during the rule blocks in the fMRI phase (see also red-yellow regions in **Fig 4A**, main text). Results are reported at a *p* < 0.05 corrected threshold at the cluster level with 50 voxels of cluster extent, with an additional uncorrected *p* < 0.005 threshold at the voxel level. MNI coordinates were used. BA, Brodmann Area.

| Anatomical area | Coordinates | Cluster Size | t-value |
| --- | --- | --- | --- |
| Left Inferior Parietal Lobe (BA 40)  Left Postcentral gyrus (BA 2) | -54 -40 32 | 924 | 6.89 |
| Left Inferior Frontal Gyrus (BA 44,45)  Left Insula (BA 13) | -54 12 2 | 936 | 6.77 |
| Right Inferior Frontal Gyrus (BA 44,45)  Right Insula (BA 13) | 44 16 6 | 545 | 5.17 |
