# Supplementary material for: Integrating when and what information in the left parietal lobe allows language rule generalization
Source: PLoS Biol. 2020 Nov 2;18(11):e3000895. doi: 10.1371/journal.pbio.3000895 (PMC7660506; doi:10.1371/journal.pbio.3000895)
Supplement: S2 Table — Group-level fMRI local maxima for areas correlating with rule effect increments in Part 2 for the rule versus no-rule contrast during the fMRI phase (see also red-yellow regions in Fig 4B). Results are reported at a FWE p < 0.05 corrected threshold at the cluster level with 50 voxels of cluster extent, with an additional uncorrected p < 0.005 threshold at the voxel level. MNI coordinates were used. BA, Brodmann Area; fMRI, functional MRI; FWE, family-wise error; MNI, Montreal Neurological Institute (DOCX) [file pbio.3000895.s006.docx]

**S2 Table. Whole brain fMRI activity related to individual differences in the rule effect increment for Part 2.** Group-level fMRI local maxima for areas correlating with rule effect increments in Part 2 for the rule vs. no-rule contrast during the fMRI phase (see also red-yellow regions in **Fig 4B**). Results are reported at a *p* < 0.05 corrected threshold at the cluster level with 50 voxels of cluster extent, with an additional uncorrected *p* < 0.005 threshold at the voxel level. MNI coordinates were used. BA, Brodmann Area.

| Anatomical area | Coordinates | Cluster Size | t-value |
| --- | --- | --- | --- |
| Left Mid/Sup. Frontal Gyrus (BA 8,9)  Left Precentral Gyrus (BA 6) | -40 26 36 | 1650 | 7.12 |
| Right Pallidum  Bilateral Caudate | 16 0 -2 | 655 | 6.09 |
| Bilateral Precuneus (BA 7, 5)  Bilateral Post/Mid. Cingulate Gyrus (BA 24, 31)  Bilateral Inf./Sup. Parietal Gyrus (BA 40, 7)  Bilateral Postcentral Gyrus (BA 3) | -14 -62 32 | 4277 | 5.90 |
| Left Postcentral Gyrus (BA 2,3)  Left Inf. Parietal Gyrus (BA 40) | -34 -22 48 | 894 | 5.85 |
| Right Mid./Sup. Frontal Gyrus (BA 9,10) | 32 34 28 | 721 | 5.48 |
