# Supplementary material for: Integrating when and what information in the left parietal lobe allows language rule generalization
Source: PLoS Biol. 2020 Nov 2;18(11):e3000895. doi: 10.1371/journal.pbio.3000895 (PMC7660506; doi:10.1371/journal.pbio.3000895)
Supplement: S3 Table — (DOCX) [file pbio.3000895.s007.docx]

**S3 Table.** Coordinates for parietal stimulation sites.

| Subject | Coordinates | | |
| --- | --- | --- | --- |
| 1 | -58 | -32 | 50 |
| 2 | -44 | -46 | 52 |
| 3 | -56 | -30 | 50 |
| 4 | -50 | -26 | 46 |
| 5 | -48 | -42 | 50 |
| 6 | -54 | -30 | 52 |
| 7 | -44 | -36 | 44 |
| 8 | -55 | -54 | 38 |
| 9 | -38 | -46 | 60 |
| 10 | -48 | -46 | 56 |
| 11 | -42 | -34 | 44 |
| 12 | -56 | -36 | 44 |
| 13 | -46 | -38 | 46 |
| 14 | -44 | -50 | 54 |
| 15 | -48 | -34 | 54 |
| 16 | -50 | -30 | 46 |
| 17 | -34 | -56 | 54 |
| 18 | -58 | -48 | 40 |
| 19 | -38 | -42 | 50 |
| 20 | -46 | -36 | 46 |
