# Supplementary material for: Integrating when and what information in the left parietal lobe allows language rule generalization
Source: PLoS Biol. 2020 Nov 2;18(11):e3000895. doi: 10.1371/journal.pbio.3000895 (PMC7660506; doi:10.1371/journal.pbio.3000895)
Supplement: S4 Table — (DOCX) [file pbio.3000895.s008.docx]

**S4 Table.** Results for the *d’* calculated with the order or the dependency as false alarms

| **Violation** |  | **Session** |  |  |
| --- | --- | --- | --- | --- |
|  | **Session 1** | | **Session 2** | |
|  | **Part 1** | **Part 2 (fMRI)** | **rTMS POz** | **rTMS IPL** |
| **d’_Ord_ CxA**  **Intervention Group** | d’_Ord_ = 0.75 ± 0.78  *t*(16) = 3.94  *p* < 0.001  d_Cohen_ = 0.95 | d’_Ord_ = 1.15 ± 1.01  *t*(18) = 5.01  *p* < 0.001  d_Cohen_ = 1.14 | d’_Ord_ = 1.10 ± 0.88  *t*(19) = 5.55  *p* < 0.001  d_Cohen_ = 1.24 | d’_Ord_ = 0.98 ± 1.15  *t*(19) = 3.83  *p* < 0.001  d_Cohen_ = 0.85 |
| **d’_Dep_ A1xC2**  **Intervention Group** | d’_Dep_ =-0.04 ± 0.51  *t*(16) = -0.30  *p* = 0.76  d_Cohen_ = -0.07 | d’_Dep_ = 0.06 ± 0.89 *t*(18) =0.32  *p* = 0.74  d_Cohen_ = 0.07 | d’_Dep_ = 0.09 ± 0.86 *t*(19) = 0.50  *p* = 0.62  d_Cohen_ = 0.11 | d’_Dep_ = 0.21 ± 0.79 *t*(19) = 1.18  *p* = 0.25  d_Cohen_ = 0.26 |
|  | **Part 1** | **Part 2** |  | |
| **d’_Ord_ CxA**  **Control Group** | d’_Ord_ = 0.75 ± 0.78  *t*(31) = 5.01  *p* < 0.001  d_Cohen_ = 0.88 | d’_Ord_= 1.05 ± 1.19  *t*(31) = 4.98  *p* < 0.001  d_Cohen_ = 0.88 | d’_Ord_= 1.25 ± 1.01  *t*(31) = 6.98  *p* < 0.001  d_Cohen_ = 1.236 | |
| **d’_Dep_ A1xC2**  **Control Group** | d’_Dep_ = 0.08 ± 0.47  *t*(31) = 1.02  *p* = 0.315  d_Cohen_ = 0.180 | d’_Dep_ = 0.37 ± 0.97 *t*(31) = 2.19  *p* < 0.04  d_Cohen_ = 0.387 | d’_Dep_ = 0.50 ± 1.08  *t*(31) = 2.62  *p* < 0.02  d_Cohen_ = 0.463 | |

When the *d’* was calculated for each type of violation separately, we observed that in all sessions and groups, participants were sensitive to order (CxA) indicating they learned the position of the dependencies (see Table S4). Indeed, learning of position was maintained despite the rTMS intervention on Session 2 and was observed in all conditions with no differences between them (rTMS POz to rTMS lPL within subjects comparison: *d’*_Ord:_*: t*(19) = 0.57, *p* = 0.57, d_Cohen_ = 0.129; rTMS POz to Control between subjects comparison: *d’*_Ord_*:*t(50) = 0.56, p = 0.57, d_Cohen_ = 0.161; rTMS lPL to Control between subjects comparison: *d’*_Ord_*: t*(50) = 0.95, *p* =0.34, d_Cohen_ = 0.271). However, only the Control group was sensitive to the dependency violations (A1xC2; see Table S4) pointing that only this group, which was tested three times with no interference in any of the three languages learned, was able to benefit from the repetitive testing to extract this more detailed knowledge of the dependencies in Session 2 (i.e. generalization session). Nevertheless, no strong conclusions can be drawn from this result since there were no differences in Session 2 between rTMS POz and rTMS lPL in the within subjects comparisons (d’_Dep_*: t*(19) = 0.59, *p* = 0.56, d_Cohen_ = 0.133) but also no significant differences in the between subjects comparisons (rTMS POz to Control group: *d’*_Dep_: t(50) = 0.98, p = 0.330, d_Cohen_ = 0.280; rTMS lPL to Control group: *d’*_Dep_*: t*(50) = 1.41, *p* =0.160, d_Cohen_ = 0.403.
