# Supplementary material for: Integrating when and what information in the left parietal lobe allows language rule generalization
Source: PLoS Biol. 2020 Nov 2;18(11):e3000895. doi: 10.1371/journal.pbio.3000895 (PMC7660506; doi:10.1371/journal.pbio.3000895)
Supplement: S1 Text — (DOCX) [file pbio.3000895.s010.docx]

**Offline recognition test**

*Methods*

Following each online learning phase, participants’ knowledge of the rules was additionally assessed via a recognition test. Participants were presented with phrases that conformed to the rules in half of the trials and phrases that violated them in the other half. Incorrect sentences were, in half of the cases, violations of the dependency using A and C elements from different rule structures in their correct position but violating the specific A_C *dependency* (i.e. A1xC2, A2xC1), and, in the other half, they were *order* violations, whereby A and C elements swapped positions within the phrase albeit maintaining their specific dependency (i.e. C1xA1 and C2xA2). Correct sentences were in half of the trials the same sentences previously presented and in the other half they were new sentences that contained an x from the pool of x exposed in the language but not previously combined with the specific A1_C1 or A2_C2 dependency. The complete offline test thus comprised a total of 48 test phrases (24 per rule dependency). Participants were instructed to discriminate between phrases that could and could not belong to the previously heard language by pressing the corresponding button. Button-response relation was counterbalanced across participants. No limits on response time were set in the baseline and rTMS tests, though participants were instructed to respond quickly after the whole phrase was heard. A new phrase was delivered immediately after a participant’s response. In the fMRI scanner, a response time maximum threshold of 1500 ms was set, along with a jittered interval between 1000 and 3000 ms before the start of the next trial.

Participants’ ability to discriminate rule items from violations was assessed by transforming to *d* prime scores (*d′*) accuracy responses. For each participant, the proportion of hits (i.e. *yes* responses to rule phrases) and false alarms (i.e. *yes* responses to violations) were used to calculate the *d′* score after taking care of hit and false alarm rates of zero or one (49). We computed three different *d’* scores by calculating the false alarms using: i) both order and dependency trials (*d’_All_)*, ii) order violations (*d’_Ord_*), and iii) dependency violations (*d’_Dep_*). These scores were then submitted to one-sample and paired *t*-tests to test for statistical significance (*d′* = 0 corresponding to no discrimination). In order to assess the effects of the rTMS lPL intervention, *d’* in this condition was compared to *d’* scores in the rTMS POz intervention in the same subjects. In addition, *d’* under the rTMS lPL effects was also compared to *d’* in the Control group as a between-subjects comparison.

*Results*

Participants from both groups were able to significantly discriminate sentences that followed the learned dependencies from all violations in all phases (Session 1 Part 1: Intervention group, *d’All* = 0.34 ± 0.44,  *t*(16) = 3.185, *p* < 0.01, d_Cohen_ = 0.773, Control group, *d’All* = 0.42 ± 0.46, *t*(31) = 5.13, *p* < 0.001, d_Cohen_ = 0.907; Session 1 Part 2: Intervention group, *d’All* = 0.55 ± 0.80, *t*(18) = 3.03, *p* < 0.01, d_Cohen_ = 0.696, Control group, *d’All* = 0.69 ± 0.92, *t*(31) = 4.24, *p* < 0.001, d_Cohen_ = 0.749; rTMS lPL: Intervention group, *d’All* = 0.54 ± 0.74, *t*(19) = 3.25, *p* < 0.004, d_Cohen_ = 0.729; rTMS POz: *d’All* = 0.55 ± 0.71, *t*(19) = 3.45, *p* < 0.003, d_Cohen_ = 0.773; Control group Session 2: *d’All* = 0.85 ± 0.80, *t*(31) = 5.96, *p* < 0.001, d_Cohen_ = 1.045). When specifically looking at the effects of the rTMS intervention on Session 2 we observed it had no effect on the *d’* discrimination scores. Performance was comparable in the rTMS lPL and the rTMS POz interventions in the within subjects comparison (*d’All: t*(19) = 0.11, *p* = 0.912, d_Cohen_ = 0.025) and there were no significant differences for the comparison of the rTMS lPL and the rTMS POz conditions to the performance of the Control group in the between subjects comparisons (respectively, *d’All: t*(50) = 1.39, *p* =0.170, d_Cohen_ = 0.397 and *t*(50) = 1.33, *p* = 0.189, d_Cohen_ = 0.379).
