# Supplementary material for: Integrating when and what information in the left parietal lobe allows language rule generalization
Source: PLoS Biol. 2020 Nov 2;18(11):e3000895. doi: 10.1371/journal.pbio.3000895 (PMC7660506; doi:10.1371/journal.pbio.3000895)
Supplement: Attachment — Submitted filename: Rebuttal letter.docx [file pbio.3000895.s016.docx]

Dear Dr. Gasque,

Thank you very much for your quick response and the explanation to your editorial decision. With all due respect to the editorial work, we would like to make this appeal to state why our work goes well beyond the state of the art moving knowledge clearly forward from what is known so far in the literature.

In general terms i) the theoretical proposals put forward so far have been based solely on behavioral research and remain agnostic concerning the biological bases of the mechanisms involved, ii) the attentional mechanisms that may be engaged are underspecified, and iii) the most important point, which is how rules become abstract, still remains to be elucidated.

In particular, going back to the work mentioned in your letter and included in our introduction, all this is valuable and important work, allowing us to build our hypothesis however this work is still far from what we achieved in our submitted manuscript. As mentioned in the editorial letter and in our introduction, it has been shown that diverting attention interferes with rule generalization (Ref 18- Toro et al 2005. Cognition) and the deployment of attention has been shown to be crucial in determining what information will be learned (Ref 13 – Lopez-Barroso et al 2016. Cognition) to bias subsequent learning (Ref 15 – Jiang and Chun 2010 QJEP). However, critically, i) none if this work specifies which type of attention is involved and how does attention enable generalization, ii) this work is only behavioural and gives no hint on what is the biological basis of this attention engagement, iii) it does not look at the temporal dynamics of learning process since they observe only the effects after learning giving little explanation of when in the learning process attention kicks in. The letter also mentions Refs 16,17 – Ellis 2006. Appl Linguist. Robinson 2017). One is a theoretical proposal and the other a review, both proposing that attention underpins the ability to transfer the rules of a first language to a structurally similar second language. These are not sustained on experimental work but are based on observations from the field of second language acquisition.

All this work comes from different backgrounds that usually do not talk to each other: second language acquisition literature and experimental behavioural research studies. We have related them in our study adding also the research from attention, clearly not considered previously, and from cognitive neuroscience to build our hypothesis on the specific mechanisms underlying learning and generalization and the biological basis of them. In our work, we give support to the involvement of attention which was earlier debated and the detailed role was not given. In particular, we specify the attentional mechanisms allowing abstraction previously unknown showing it is the interaction between attention to what is expected and attention to when this is expected that allows later generalization. We also give a biological basis that sustains this mechanism showing that the left parietal lobe is causally related to rule generalization due to its role in the integration of what and when information. We also detail the dynamics of the learning process showing how statistical learning and attention arise at different moments of the learning process and the networks involved at each stage.

In sum, from what we have outlined here there is currently a true need to clarify the mechanisms underlying language rule-learning and the way they play themselves out at a neural level going beyond a descriptive level. Here, we combined behavioral, functional neuroimaging and transcranial magnetic stimulation and integrated recent findings on attention to a) show that language rule learning is a two-stage process and b) specify the attentional mechanisms underlying rule abstraction and c) provide their neural support. As we mentioned in our letter and we hope it is clearer now, by taking a different perspective from previous work - giving an explanation at the intersection of systems and cognitive neuroscience, language learning and attention research – our findings go beyond the state of the art in the field. Based on theoretical work from different fields based solely on behavioural research we here built a specific hypothesis and give support to it specifying the dynamics of the learning process and their neural sustain. In addition, we give support to the involvement of attention which was earlier debated and the detailed role was not given. In particular, we specify the attentional mechanisms allowing abstraction previously unknown showing it is the interaction between attention to what is expected and attention to when this is expected that allows later generalization. This is a major turnover in the field. Moreover, the findings have implications for neural mechanisms and behavior that extend well beyond the immediate topic of the work we present since they can apply to the mechanisms of rule abstraction outside the language domain. We believe our cover letter may not have highlighted these important points so that you could appreciate the value of our work for your journal.

We thank you for your time and efforts.

Sincerely,
