# Supplementary material for: Integrating when and what information in the left parietal lobe allows language rule generalization
Source: PLoS Biol. 2020 Nov 2;18(11):e3000895. doi: 10.1371/journal.pbio.3000895 (PMC7660506; doi:10.1371/journal.pbio.3000895)
Supplement: Attachment — Submitted filename: Orpella et al Plos Bio_Response to Reviewers.docx [file pbio.3000895.s018.docx]

First of all we would like to thank all reviewers for their thorough reviews and extremely helpful suggestions that have with no doubt led to greatly improve the manuscript. We hereby give a point by point answer to the questions they raised. The additions and changes in the manuscript are highlighted in red with a mention here to the exact pages and lines where these changes can be found.

Reviewer comments:

**Reviewer #1, Jutta L. Mueller: The manuscript reports a multimodal study on incidental non-adjacent dependency learning with adult learners undergoing behavioural tasks, fMRI scanning and rTMS stimulation. The learning task comprises the learning of non-adjacent dependencies between two bisyllabic units which are presented in separated three-unit phrases. A word monitoring task was used to measure incidental learning. Functional brain activation was evaluated during learning using different learning-related measures as parametric regressors. The authors identified the ventral and dorsal attention network comprising parietal and frontal regions as related to different aspects of learning. Subsequently rTMS applied to the involved SPL region selectively modulated the application of previously learned rule-knowledge to new material.
The investigation of on-line rule-learning processes in a multimodal setup is a timely and interesting approach to unravel the brain mechanisms involved in language acquisition. Specifically, the inclusion of rTMS in order to investigate the causal contribution of a specific brain region is great. While I applaud the general approach I had severe upcoming doubts regarding the validity of the results as I was reading through the manuscript in more detail. My two main concerns are i) the framing as a paradigm testing statistical learning and, more seriously, ii) the claim to test the acquisition of non-adjacent dependencies, which is, as far as I can see now, not unambiguously shown by the reported behavioral tests. As these two points concern core aspects of the paper, I recommend substantial revisions before for a potential publication.**

*We want to thank the reviewer for her positive comments about our work and for applauding our multimodal approach, especially the inclusion of rTMS. We also want to thank her for her revision, which has certainly improved the quality of this manuscript. Below, the reviewer will find point by point answers to her comments addressing all her concerns.*

**Major

1. General framing: In their general framing the authors juxtapose statistical learning to attention-based rule learning and claim that their paradigm taps into both processes with statistical learning in initial stages and goal-directed attention in the later stages. While it is difficult to disagree that both processes play a role during the acquisition of complex rules, I have some difficulties applying the concept of statistical learning to the present paradigm. The three-word phrases are presented with jittered pauses during learning as far as I understood the paradigm. This means that there were additional perceptual cues to the to-be-learned structure from the start (namely pauses between the phrases with the crucial elements at the edges). Statistical learning, as it was introduced by Saffran et al., referred to the learning of transitional probabilities between units of speech without any additional acoustic cue. As soon as additional cues are provided learning may not be purely statistical anymore, but guided by other principles of perception. I agree that goal-directed attention must play a larger role as learning proceeds but in my view it is incorrect to subsume the processes before that under the umbrella of statistical learning.**

*We understand the view of the reviewer regarding Statistical Learning (SL) and we do agree, in hindsight, that we should have better explained how SL encompasses a broader concept and mechanism. We, however, respectfully disagree—based on data from multiple studies across labs and domains—with the reviewer’s main concern that SL refers only to the learning of transitional probabilities without any additional acoustic cue. Research on this particular aspect (i.e., learning from a continuous stream of stimuli) is only a fraction of what the study of SL is. Statistical Learning is broadly defined as the ability of humans and other animals to extract statistical regularities from the world around them. Even in the restricted case of such abilities within language, taking Jenny Saffran’s words: “it capitalizes on the statistical properties of the language environments, including the distribution of sounds in words and the orders of word types in sentences” (Saffran, 2003). Indeed, Dr. Saffran’s own research on statistical learning (to which the reviewer refers in her comments), has also tackled the learning of syntactic dependencies (Saffran and Wilson 2003) using a very similar paradigm to the one we used in our work, i.e. segmented words organized in 3-word sentences. Just to give more examples aside from Saffran’s work, SL has also been studied in the context of word-referent matching (Yu and Smith 2007), and outside the language domain in terms of contingencies between pairs of images (Richter and deLange 2019) or between distractor and target locations (van Moorselaar and Slagter 2019). None of these studies (including that of Saffran and Wilson, 2003) presented continuous streams of stimulation. Instead, they all used, as in the case of our study, trials with jittered pauses between them; and all of them fall within SL.*

*Moreover, the reviewer in her statement conflates SL and the computations underlying SL (presumably, the tracking of transitional probabilities). These are two different things. To make the statement clearer, it is interesting to compare SL in humans and animals. Research in rats indicates they do have SL abilities but use different underlying computations than those employed by humans: while humans track transitional probabilities, rats use frequency of co-occurrence (Toro et al., 2005).*

*The other aspect that underlies the reviewer’s comment is the fact that there are other principles of perception that can add to SL (note that it does not mean that SL mechanisms are not used, but that other principles can additionally be at play). This issue comes, among others, from the proposal by Endress, Nespor and Mehler (2009). Indeed, Endress and collaborators suggest that the use of pauses introduces edges that can be (in their words) “used as anchor points for extracting positional regularities”. We do not deny that pauses are relevant and play a role (as they do in real life language learning), but regularities still need to be extracted in this situation, as the authors acknowledge in their own work (Endress, Nespor and Mehler, 2009). In our case, the gradual effects observed in the results of the online behavioral measure in early learning support the fact that statistical learning is taking place more than a simple perceptual effect due to the presence of pauses/edges. This latter mechanism may be more relevant for the generalization phase where these pauses/edges provide the temporal anchor points that can be used to replace the content of the previously extracted dependencies. This aspect is actually already mentioned in the discussion and in the new version of the manuscript is further emphasized.*

*In all, we acknowledge that this is an interesting debate and we have modified our introduction (p. 3, lines 32-43) to clarify the points raised by the reviewer. This now reads:*

*“Given its core relevance, the mechanisms supporting rule learning have been subject to intense debate. By their very nature, language rules are characterized by sequential co-occurrences, often between non-adjacent elements (e.g.* ***These*** *cupcake****s*** ***are un****believ****able****). Accordingly, tracking statistical relations is known to be a key computation in this context(5) as it is in many other domains(6,7). Yet, statistical learning alone cannot fully account for the generalization of non-adjacent dependencies(8–10), and an additional role for attention has been posited as necessary(11–17). The deployment of attention has been shown to be crucial in determining what information will be learned(16) and to bias subsequent learning(18). Indeed, it is often the case in natural languages that dependencies occur at salient positions such as edges, with edges acting as perceptual anchor points that could additionally help to learn the positional information of the related elements(19). In line with this, attention has been also proposed to underpin the ability to transfer the rules of a first language to a structurally similar second language(19,20) while diverting attention interferes with rule generalization(21).”*

*In all, we believe -based on the work of multiple labs, including ours and Dr. Saffran’s- that the points raised by the reviewer clearly do not invalidate our study or the theoretical background of this work, as her major concern is based on a narrow interpretation of SL.*

**2. Another point, which is potentially more related to writing and less to actual content is the argument about the what and when information. How exactly does the current learning task measure the binding of what and when information? In the generalization task the what is new and the when is the same as in the previous learning phases. Looking at that task alone – couldn’t one argue that the when is disturbed by rTMS and for this reason the rule (relating to when) cannot be generalized to the novel items? The parietal lobe might be not necessarily involved in integrating the what and when information in the current study, but rather only in supporting the when information.**

*The reviewer nicely summarizes what happens in the generalization task: the* what *is new and the* when *is the same as in the previous learning phases. What we hypothesize is that, in order to generalize, one needs to replace the* what *previously learned and integrate it with the same* when *position. The reviewer is completely right in that, from the word monitoring task alone, the results are equally compatible with the interpretation of the rTMS intervention affecting only the* when *information. However, if this were the case, participants would have learned the association between the new A and the new C with no temporal information. This would be reflected in the offline test as a high false alarm rate for test trials in which participants need to discriminate between AxC and CxA. This is not what we observe. Participants show sensitivity to the positional information of the dependencies and this temporal information is not affected by the rTMS intervention. Following point 4 of the reviewer’s comments also, we have now detailed the results of the two types of trials (CXA and A1XC2) of the offline test in the Supplementary Information (Offline Recognition Test section and Table S4).*

*Note that, from the rTMS results of the language task, we do not conclude that what we observe is an interference in the ability to integrate* what *and* where *information. This conclusion comes from the rTMS results in the attention task. Our conclusion from the language task is that rTMS on the left parietal lobe interferes with the ability of the subjects to generalize from their previous knowledge. With respect to this task we believe also that the changes in the analysis and presentation of the results to respond to point 2 of reviewer #2 make now clearer that the interference while stimulating lPL is specific to the condition where* what *and* when *information are presented concurrently.*

**3. Experimental setup: In the learning phases rule-blocks comprise only rule phrases and no-rule blocks comprised different combinations of bisyllabics. Non-adjacent dependency learning is assessed by a word monitoring task where participants are asked to respond to the final word which can be either predicted (in rule blocks) or not predicted (in no-rule blocks). I have a serious concern with regard to the validity of the task. How do we know that it measures non-adjacent dependency learning? From the information in the manuscript it is unclear to me with which frequency the final words occur across the rule and no-rule blocks. As I understand it the final words must be more frequent (independently of the non-adjacent dependency) in the rule blocks compared to the no-rule blocks in which all sorts of elements can occur at the end of a phrase. If the base frequency at the final position for a specific word is increased in the rule blocks it cannot be known anymore whether the RT effect in the word monitoring task is related to mere frequency of that word in the respective position or to the acquired non-adjacent dependency. This means that the rule that was learned could have been much more simple than suggested, namely only that “there is a 50% chance that the target word occurs at the final position” in contrast to “there is a 25% chance that the target word occurs at the final position”. From the manuscript it was unclear to me how exactly the different items are distributed across the blocks. This should be clarified and item lists should be made publicly available (also for replication purposes). If my concern results from a misunderstanding of the design I am happy to accept word monitoring as a measure of incidental non-adjacent dependency learning. But this can hold only when the positional base frequencies of the words were kept constant across blocks.**

*We thank the reviewer for pointing out the need for a clearer description of the rule-learning task design. We acknowledge that the way the stimuli were described in the methods section may give rise to some confusion. When we described the no-rule condition we used the term “fillers” to introduce the combination of XXX elements and this may have created some confusion in relation to the frequency of occurrence of the target word. However, we would like to emphasize that the probability of target and, therefore, of no target appearance in each string is 0.5* in both *the rule and the no rule blocks. Thus, RT effects reported for the rule blocks compared to the no rule blocks can only be explained in terms of the learning of the non-adjacent dependencies, which is only possible in the rule blocks. To make this important point clearer in the manuscript, we have now rephrased the way the no rule condition is explained removing the term “filler” which was misleading. The corresponding methods section (lines 568-580) now reads:*

*“A total of 96 rule and 96 no-rule phrases (trials) were used in this task. Rule phrases conformed to an AXC structure whereby the initial word (A) always predicted the final word (C), while the middle word (X) was variable. Two different A_C dependencies (A1_C1 and A2_C2) were created out of 4 words from the total word pool. The remaining 24 served as middle (X) elements for each of the two A_C dependencies. The transitional probability was always 1 between A and C elements, 0.04 between A and X, and 0.5 between X and C. Half of the no rule phrases consisted in the combination of 3 of the 24 X elements and so took the form XXX, with the only constraints that each X had an equal probability to appear in each position but could never appear twice in the same phrase. The other half of the no-rule trials consisted of the combination of two XX elements (following the same constraints as for XXX) followed by the participant’s target word (C1 or C2). The probability of target occurrence in both the rule and the no-rule blocks was therefore 50%. Note that in the set of no-rule trials, the C element (the participant’s target) occurred also in the last position but, in contrast to the rule block, this could not be predicted on the basis of previous elements.”*

*We have also rewritten one sentence in the main ms to make the description of the materials clearer (Page 7, lines 127-129): “In no-rule blocks, three-word phrases with no dependencies (XXX-type and XXC-type strings) were used. Each three-word phrase was considered a trial.*

*The reviewer’s major concern regarding the frequency of the target occurrence in the rule and no-rule conditions is therefore dispelled by this clarification of the stimuli set. We also note that a similar word-monitoring task was used in López-Barroso et al. (2016) to assess incidental non-adjacent dependency learning. We have additionally included a full list of the stimuli employed in the task (Supporting Information Table S5) for review and replication purposes.*

**4. Relating to this point I am not sure what exactly the offline recognition test measured (and also when exactly it was done – this should be included in the figure (Fig. 1) of the experimental setup).The authors report that three types if test items were used, correct (A1xC1), non-adjacent dependency violations (A1xC2) and swapped (C2xA2). Only the second type of items is useful to assess whether specific non-adjacent dependencies were learned. But the results for this item type are not reported separately and it is not known whether the d’ scores are solely driven by the discrimination of the first and third item type or whether the non-adjacent dependencies were learned as well.**

*The reviewer raises an important point here that we should have better emphasized in the previous version of the manuscript. The offline test assessed the learning of the dependencies, in other words, whether participants were not only implicitly sensitive to the dependencies but also retained a more precise knowledge of the A_C structure of the language (see also Lopez-Barroso et al 2016 where a similar approach was adopted). An offline test is the measure most commonly used in the literature to assess this kind of learning and was therefore included in our paradigm for completeness. However, in our study, this was not our main interest and was only employed to test general learning of the dependencies. The novelty of our approach relies on the fact that we have an online measure of learning that allows us to track how participants are progressively able to predict the elements of the dependency. That is why we did not include the details of the different types of trials for the offline test in the supplementary information. To put it in a nutshell, our work focuses on the online learning and generalization of the non-adjacent dependencies.*

*The different test items used measured different aspects of non-adjacent dependency learning. This allowed us to compute a summary offline measure: A1xC1 tested whether participants recognized the specific phrases presented in the learning phase; A1xC2 tested whether they learned the specific relations of A1-C1 and A2-C2 or they only learned that As appeared in initial position and Cs in final positions; CxA tested whether they learned the relations irrespective of the order and thus irrespective also of the temporal information. Coming back to point 2 of the reviewer, this last test items should trigger a high false alarm rate if the temporal information is not considered in the dependency rule. Our results in fact show the opposite pattern: d primes for participants from both groups at any session are significantly above chance when using CxA test trials to compute the false alarm rate. We agree with the reviewer that calculating d primes using the different test trials will enrich the paper and we have included this information in the supplementary information section (Offline Recognition Test section, and Table S4). We have also included reference to the offline test in Fig. 1.*

**5. What was the method of correction for multiple comparisons in the fMRI part? How was the minimal cluster size (50 voxels) determined? Please add that information to the manuscript**

*We used a p < 0.05 FWE-corrected threshold at the cluster level, with an additional p < 0.001 at the voxel level. While this information was provided in the legend of Figure 4, we did not include in the methods that the correction used was FWE. We thank the reviewer for catching this and have corrected the manuscript accordingly (page 32, line 709). The cluster size was selected to be roughly double of what has been previously recommended to be a voxel extent that produces a desirable balance between Types I and II error rates (Lieberman and Cunningham, 2009).*

**6. Please make the whole list of stimuli available.**

*The full list of stimuli for the incidental rule-learning task is now available in the Supporting Information section of the manuscript (Table S5).*

**Minor

7. Page 6: mean age = 23.63 --> 23.63 years, please add also for other age specifications.
Page 14: latter --> later.**

*Thank you for catching these minor points. We have modified the manuscript accordingly.*

**8. What is “posterior generalization”?**

*The adjective* posterior *was used to point out the staged nature of rule learning, with a first stage where the rule is learned and abstracted from the specific instances, and a subsequent (posterior) stage where this knowledge can be generalized to learn new instances of the rule. We have now changed this to ‘*subsequent generalization’ *in line 495 of the manuscript in the hope that this more faithfully reflects the idea.*

**9. Page 16: What exactly is a trial? Is one trial a three-word phrase?**

*Indeed, we refer to the presentation of each a three-word phrase as one trial. To make this clearer, the manuscript now reads:*

*(p. 7, lines 123-127) ‘In rule blocks, participants were exposed to three-word phrases containing AXC-type dependencies with the first word A predicting the last word C irrespective of the intermediate word X (e.g. ‘jupo [variable word] runi’). In no-rule blocks, three-word phrases with no dependencies (XXX-type and XXC-type strings) were used. Each three-word phrase was considered a trial. Rule and no-rule trial blocks were interleaved (see Methods).’*

*(And p. 27, line 568) ‘A total of 96 rule and 96 filler phrases (trials) were used in this task.’*

**Reviewer #2: Orpella and colleagues combined an implicit artificial language learning task with fMRI and inhibitory transcranial magnetic stimulation (TMS) to investigate the role of the left inferior parietal lobe (IPL) in goal-directed attention during language rule generalization. First, healthy subjects underwent a behavioral language learning paradigm insight the MR scanner (or outside for the control group). Thereafter, the fMRI group received inhibitory TMS over either left IPL or a control site and performed learning tasks on two different language sets and completed an attention paradigm. Results were compared within-group and with a control group that did not receive TMS. In the behavioral data, the authors observed a shift from statistical learning to goal-directed attention in later phases. This shift was underpinned by the recruitment of a broad dorsal attention network, including the IPL. TMS over the left IPL impaired the subjects’ ability to generalize
learned rules to a structurally analogous new language as well as the ability to integrate stimulus identity and timing in the attention task. Based on these findings, the authors conclude that language rule generalization is a two-step process that engages statistical learning and goal-directed attention, with a key role of the left IPL to integrate stimulus information about identity (what) and timing (when).**

**This is a potentially interesting paper that uses a multimodal combination of different approaches (behavioral learning, fMRI, TMS) to tackle the role of the IPL in language rule generalization. While the approach is interesting, I have several major issues that limited my initial enthusiasm about the present study**.

*We really want to thank the reviewer for his/her work and for the positive comments regarding our multimodal approach. Below, the reviewer will find point by point answers to all his/her comments. The manuscript has greatly benefited for this reviewer’s insights and we hope that this improvements renew the reviewer’s initial enthusiasm for our work.*

**1. One major issue with the design was that the control group did not receive any TMS intervention (and no fMRI) and should thus not really be considered as a control group. Why didn’t you include sham stimulation here? Without a sham condition, it is hard to judge whether the observed effects result from a decrease in accuracy after IPL TMS or might also be influenced by a potential facilitation for the active control site. No TMS cannot be regarded as adequate control site because there was simply no intervention, and no side effects.**

*We apologize for a possible lack of clarity in explaining our control measures and we hope our clarifications resolve the reviewer concerns. We had very clear reasons for using a control site stimulation (rTMS POz) and a Control group without any intervention rather than a Sham stimulation. The rTMS stimulation on a control site was a better control than a Sham condition since it was better matched to the rTMS-lPL intervention (i.e., active rTMS stimulation). A difference between those conditions cannot be explained by an unspecific effect of rTMS stimulation and can only reflect an effect specific to the site stimulated. A possible concern may then be that rTMS-POz could show an effect as well. The comparison of this condition with the Control group with no intervention whatsoever is a perfect comparison to show the rTMS-POz stimulation had no effect at all.*

*Therefore, we respectfully believe that the reviewer’s major concern is unfounded when we consider: i) that the purpose of the behavioral Control group was to provide a measure of the repetition effects of testing language learning with several languages on rule generalization in baseline circumstances; and ii) that all behavioral measures used during session 2 (slopes, Rule Effect, d prime for the offline test) did not differ between the Control group and the intervention group during rTMS POz. As our manuscript reads (p. 6, lines 109), the Control group was included to assess task repetition effects in the rule-learning task; in other words, to provide a baseline for the effect of rule generalization precisely without any form of intervention (fMRI or rTMS). We have now emphasized this at several additional points throughout the ms.*

*Page 24, lines 508-510: “The Control group was crucial to characterize the course of events under repeated exposure to the same language task and to have a baseline measure of generalization effects under no intervention.”*

*Page 13, lines 263-266: “Participants in the Control group were also tested in Session 2 with the same new languages (L2 and L3), just like the Intervention group, but without rTMS (Control group Session 2, see Fig. 1). Their performance was used to measure the effects of task repetition and to have a measure of the baseline levels of generalization without rTMS.*

*We feel that showing that the performance of the Control group is comparable to that of the Intervention group during rTMS POz legitimizes the suitability of this condition (rTMS POz) as a control rTMS site while at the same time controlling for repetition effects during rule generalization. Indeed, in the previous version of the manuscript we reported non-significant differences between the slopes of the Control group and the rTMS POz condition. We have now added the same analysis for the rule effect which shows the same results. This strongly suggest that rTMS POz had no effect (facilitatory or otherwise) on rule generalization as the reviewer hypothesized. We further emphasize this in the manuscript in the appropriate places (p. 14, lines 275-279) as:*

*“Indeed, both the rule effect and the rule slope under rTMS POz were comparable to those of the Control group (respectively, t(50) = 2.85, p = 0.271, d_Cohen_ = 0.317 and t(51) = -0.79, p > 0.4). This indicates that stimulation to POz did not influence task performance (i.e., the Control group and the Intervention group during rTMS POz have comparable slopes and rule effects) and thus confirms rTMS POz as a suitable control site for participants’ performance under rTMS lPL.”*

*As aforementioned, this result validates the reported comparisons between rTMS lPL and rTMS POZ, which we further complement with comparisons between rTMS lPL and the Control group (p. 15, lines 290-292): “The slope for rule blocks in this phase was significantly more negative than both the slope after rTMS stimulation to the control site POz (ßdiff = -0.67, t = -2.66, p = 0.008) and that of the Control group Session 2 (ßdiff = -0.57, t = -2.49, p = 0.013)”.*

**2. On a related note, I was surprised that the analyses never directly compared TMS over both sites. I would have expected that the step-down procedure would include a direct comparison of TMS over IPL and the control site (for both the language task and the attention task), but it didn’t. How can you draw strong conclusions if you never directly contrast IPL TMS and control site? Just because IPL TMS disrupts performance and control stimulation doesn’t impair accuracy, it doesn’t mean that the effect for IPL is larger than the effect for the control site. I think this is a main problem with the current study. Direct comparisons are needed here to draw valid conclusions.**

*We are very thankful to the reviewer for this suggestion. We agree with the reviewer that direct comparisons between rTMS IPL and rTMS POz are crucial to ensure that the reported effects are valid. That is why direct comparisons between rTMS conditions were explicitly reported for the rule-learning task in the previous version of the manuscript revised by this reviewer (pp. 12-13). As for the attention task, we acknowledge that there are different ways to break down and present complex interactions such as those emerging from this task. In writing the manuscript, we chose the way that, in our view, provided the clearest and most intuitive picture of the rTMS lPL effects. In this respect, we report a main effect of rTMS intervention, a three-way interaction between rTMS intervention, Rhythm and Identity, and interactions in opposite directions between Rhythm and Identity in rTMS POz and lPL. These interactions are then explained in the context of each rTMS intervention. Having detailed this, it is true that the suggestion of the reviewer to choose a different breakdown procedure allowing direct comparison of the effects in the two stimulation sites is definitely more convincing. We have therefore now changed the way we have analyzed the results in the stepdown procedure to allow for this direct comparison. For this we have looked at how the use of the informative cue about the identity of the target (what information) varies as a function of whether the temporal information is predictive or not under the two rTMS interventions. For this we have run ANOVAs with the within subject factors Intervention (rTMS POz, rTMS lPL) and Identity (Informative, non-informative) separately for the Rhythmic condition and Non-rhythmic condition. The results and conclusions do not vary, as expected, but the writing and effects are now much clearer and straightforward. We really appreciate this suggestion, which has greatly improved this important part of the manuscript. We have also changed the figure 7 to represent more clearly the breakdown procedure used in the new analyses.*

*The results of the attention task now read as follows (pages 17-18, lines 344-370):*

*“In order to test whether what and when cues where used differently in the two intervention conditions we performed a 2x2x2 repeated measures ANOVA on reaction times to the target syllable (correct responses only), with Intervention (rTMS_lPL, rTMS_POz), Rhythm (rhythmic, non-rhythmic) and Identity (informative, non-informative) as within-subject factors. This analysis revealed a main effect of Intervention (F(1,18) = 13.36, p = 0.002, Partial η² = 0.426), with overall reaction times slower after rTMS to the lPL compared to rTMS POz. Regarding the use of when cues, pitch discrimination in rhythmic sequences was faster than in non-rhythmic sequences (main effect of Rhythm: F(1,18) = 24.62, p < 0.001). Concerning the effect of what cues, there was also a facilitation in reaction times for informative compared to non-informative sequences (main effect of Identity: F(1,18) = 9.01, p = 0.008). The main effects of Rhythm and Identity thus indicated that the attentional manipulations were effective and participants benefited from the use of both what and when information. Crucially for our hypothesis, we observed a significant triple interaction between Intervention, Identity and Rhythm (F(1,18) = 7.54, p = 0.013, Partial η² = 0.295), not affected by the order of rTMS intervention (F < 1), suggesting differences in how when and what cues were used under each rTMS intervention (Fig. 7). We then proceeded to unpack the interaction by analyzing how information about what was affected by the presence or absence of predictive information about when the target is expected in each rTMS intervention. In sequences in which when information was unpredictable (non-rhythmic sequences), Identity had a comparable facilitatory effect in both rTMS sites (Identity: F(1,19) = 9.58, p = 0.006, Partial η² = 0.335; Intervention x Identity: F(1,19) = 1.12, p > 0.3, Partial η² = 0.056). However, in sequences in which when information was predictive (rhythmic sequences), Identity had a facilitatory effect (Identity: F(1,19) = 4.64, p = 0.044, Partial η² = 0.196) that differed in the two rTMS stimulated sites (Intervention x Identity: F(1,19) = 13.18, p < 0.002, Partial η² = 0.410). While participants could benefit from knowing what the identity of the target was while stimulated at the control site (rTMS POz, t(19) = 5.51, p < 0.0001, d_Cohen_ = 0.596), this facilitation disappeared under rTMS lPL stimulation (t(19) = -0.22, p > 0.8, d_Cohen_ = 0.027). These results indicated that while able to benefit from the what information in isolation, participants were impaired in their ability to benefit from it when both* what *and* when *cues were presented in* conjunction*.”*

**3. It also wasn’t clear to me what the interval between the two TMS sessions was – I hope they were not performed on the same day (potential carry-over effects)?**

*We thank the reviewer for this comment. It might not have been sufficiently highlighted that rTMS interventions were of course counterbalanced and the results reported were not affected by order. Thus, yes, both rTMS interventions were indeed carried out on the same day (now explicitly specified in the manuscript:*

*Page 6, lines 102: “Participants performed two rTMS conditions on the same day (Session 2)” as well as in the legend of Figure 1 (lines 100-101: “rTMS intervention order (lPL/POz) was counterbalanced between participants in the Intervention group and performed on the same day).*

*Page 6, lines 114-116: “In Session 2, participants in the Intervention group (top row, Fig. 1) received repetitive transcranial stimulation (rTMS) on the peak of activation within the left parietal lobe (lPL), and on a control site (POz), successively. Participants learned the new languages under the effects of the stimulation of each site. The order of stimulation was counterbalanced across participants.”*

*However, we note that the lack of rTMS intervention order effects (detailed on p. 15, line 299 for the rule-learning task, and on p. 18, line 356 for the attention task) rules out potential carry-over effects. In addition, here again the Control group is also helpful to show that there are no carry over effects of the rTMS interventions. The effects in this group that does not receive any rTMS intervention do not differ from the rTMS POz phase.*

**4.TMS results: Couldn't the effects be partially driven by the “no rule” condition, as there is some variation here?**

*We thank the reviewer for this suggestion. We now report, in p. 15, lines 293-297 of the manuscript, the comparison between the no-rule slopes for the different conditions and groups. The results clearly show no difference between the no-rule slopes, giving a stronger support to our results. This section now reads:*

*“Note that the effects of rTMS on the lPL were not driven by the performance in the no-rule blocks: there were no significant differences between the slopes for the no-rule blocks in rTMS lPL and rTMS POz (ß_diff_ = 0.29 , t = 1.17 , p = 0.24), rTMS POz and Control group Session 2 (ß_diff_ = 0.21, t = 0.94, p = 0.35), or rTMS lPL and Controls group Session 2 (ß_diff_ = -0.08, t = -0.33, p = 0.74).”*

**5. The strongest effect appears to be that the control group has much longer RTs. What could be an explanation here?**

*We thank the reviewer for pointing out this detail. We attribute the slower pattern of RTs for the Control group to the use of ordinary computer keyboards as response keys for testing in the University testing rooms instead of the lower latency equipment used in the rTMS facility and/or to non-specific rTMS effects (e.g. Duecker et al. 2013). We have commented on this detail in the manuscript, which now reads:*

*“Finally, we note that the overall faster RTs for rTMS conditions compared to RTs for the Control group may be due to non-specific rTMS effects(35) and/or the lower latency equipment employed for testing in the rTMS facilities and does not affect the main outcome” (page 15, lines 299-302)*

*Beyond this, we note that the main focus of the manuscript is to compare the slopes and mean RTs of rule and no rule blocks, and the slopes of rule blocks across experimental groups (rTMS POz, rTMS lPL, and Control) rather than overall latencies between experimental groups.*

**6. Were the effects corrected for multiple comparisons?**

*We refer here the reviewer to response 5 to reviewer 1. We used a p<0.05 FWE correction at the cluster level.*

**7.Why did you use mixed models for the initial behavioral analyses and ANOVAs for the TMS data? This seems a bit arbitrary.**

*We thank the reviewer for this comment that allows us to provide a clarification of the rationale used for the selection of our statistical analyses. We used mixed models when slopes were to be compared, and ANOVAs where comparing means across rule and no rule conditions (i.e. so-called rule effects). We emphasize that this is so* both *in the initial behavioral analysis* and *for the rTMS data (page 9 and page 14, respectively). The use of mixed models to compare the slope between conditions allows to use the RTs for all trials and subjects, which results in a more sensitive measure of the online learning process than a single mean value per subject (Karuza et al. 2013; Siegelman et al. 2017). Note that we could have applied an ANOVA using the* ßdiff *value of each subject but this would not be accurate since when two slopes are not significantly different, it does not make sense to detail their (mixed model) estimated* ßdiff *value, as this can be considered to amount to 0. In contrast, the comparison of mean RTs of rule and no rule conditions is suitable for an ANOVA as the Rule Difference provides a summary measure of learning when there is no slope difference between conditions and the statistical learning process is therefore finished.*

**8.The authors largely ignore the network perspective. While they acknowledge that rule learning and generalization (and attention) engages large-scale fronto-parietal networks, they selectively focus on the left IPL and do not discuss or investigate the interaction between these regions that seem to be crucial for the investigated process of interest. I found it very unfortunate that the fMRI data was simply used to localize the TMS site while the interesting interactions within and between these large-scale networks were ignored.**

*The reviewer raises an interesting point here. This is indeed the focus of another study we are presently developing. We note, however, that the current study was circumscribed to investigating the role of left parietal lobe in rule-learning and generalization. This area was specifically chosen for its involvement in temporal orienting, as noted in the manuscript. An investigation of the interactions within and between large-scale networks, however interesting, is beyond the possibilities of the current data, which was limited in the sample size for a full-fledged fMRI analysis (while adequate for rTMS and comparable to other rTMS studies; see response to point 10 below). We noted this in the manuscript by stating that: ‘*due to the limited number of subjects, these [*fMRI*] results can only be interpreted tentatively’ *(p. 13, line 247).*

**9. How did you differentiate between learners and non-learners?**

*This may have been a misunderstanding. In our study the whole group of participants is considered a continuum and we do not differentiate between learners and non-learners in any of our analyses. If the reviewer refers to the fact that we mention that in the intervention group there should be learners that are slower, this was only in relation to the observation of a slope difference while learning in the MRI. The fact that there was still a slope during MRI, suggests that some participants might have been still in the earlier stages of learning where statistical relations are still tracked (i.e., the slope is still not flat and the rule difference is not maximum). The question suggested by the reviewer is out of the scope of this work and requires a much larger sample.*

**10. How was the sample size determined?**

*We thank the reviewer for pointing out we did not detail how we determined the sample size. This is an important aspect to mention. Since there was no comparable study using TMS and a similar paradigm, the sample size was determined based on previous studies applying TMS to the parietal cortex. The sample size in these studies is between 12 subjects (e.g. Harris et al 2008, Exp 1; Ruzzoli and Soto-Faraco 2014) and 18 subjects (e.g. Marigold et al 2019) with some studies reaching 24 participants (e.g. Harris et al 2008; Capotosto et al 2012). The initial sample size was set to 22 with a final sample size of 20 that was thus expected to be largely sufficient considering that we were the in the range of the maximum sample size used the TMS studies and the main comparisons were within subjects for the rTMS intervention. This is now explained in the methods section (pp. 24, lines 510-516):*

*“Since there was no comparable study using TMS and a similar paradigm, the sample size was determined based on previous studies applying TMS to the parietal cortex. The sample size in these studies is between 12 subjects (e.g.(61,62)) and 18 subjects (e.g.(63)) with some studies reaching 24 participants (e.g.(61,64)). The initial sample size was set to 22 with a final sample size of 20 that was thus expected to be largely sufficient considering that we were the in the range of the maximum sample size used the TMS studies and the main comparisons were within subjects for the rTMS intervention*.”

**11. There is a large difference between the numbers of participants in the different groups – what was the reason for this difference?**

*As explained in point 10, from the original sample we selected 22 participants to undergo the rTMS intervention part and that sample size was chosen based on previous research.*

**12. On a related note, can one really consider the performance insight and outside the scanner as equal (intervention group and control group)?**

*The goal of the inclusion of the Control group is indeed to test for this. Since testing in the scanner and then within a rTMS protocol could have undesired effects that are not the aim of the study, the control group was included to have baseline effects of learning with no influence of fMRI or rTMS environments. The results we report show how the effects were the same for the Control group and the rTMS POz intervention for the slopes and rule effects*.

**13. It is stated that two participants from the intervention group were excluded (final sample N=20) because they were stimulated > 2 mm from the appropriate coordinate within the IPL. I don’t understand this, what does that mean? If you have identified the coordinates individually, then why were these inappropriate? With a TMS resolution of approx. 2 cm, I would not consider 2 mm as an exclusion criterion. This all seems a bit arbitrary.**

*We understand the reviewer’s concern and apologize for the lack of clarity concerning this matter. The TMS coordinates for the 2 excluded participants were not correctly stimulated due to an experimenter error, therefore rTMS was applied in an area off of the intended one. We preferred to be conservative in our exclusion criteria. Being the error in calculating the IPL coordinates a human mistake we believed that we had to remove that source of noise, since the TMS procedure in the 2 excluded participants was contaminated and therefore deviated from the rest of the cohort. We have more explicitly stated the reason for this strict criterion (p. 25, lines 530-533):*

*“Two participants from the Intervention group were excluded (final sample N=20) because they were stimulated > 2 mm from the appropriate coordinate within the lPL during rTMS (see Repetitive TMS protocol section below) due to an Experimenter error.”*

*And in the Methods (p. 33, lines 734-735): “Due to an Experimenter error, two subjects were stimulated beyond 3 mm from the peak of activation and were thus excluded from rTMS analyses*

**13. The authors largely ignore the large previous literature on artificial grammar learning and the role of the IFG by A. Friederici’s group. While some papers are cited, they are not well integrated or discussed in the discussion section. I think the paper would benefit from including them and discussing similarities and differences between the present study and the previous work.**

*We thank the reviewer for catching this and we apologize for not discussing previous literature on the role of the IFG by A. Friederici’s group more extensively. We have now included the following paragraph in the discussion of the paper (pp. 21-22, lines 432-443):*

“*Regarding language learning, several studies have previously related regions of the ventral network, such as the inferior frontal gyrus and parietal lobule, to statistical word(51–53) and rule learning(13,28,30,31,49,50,55,56). These studies have significantly added to a wealth of literature implicating the IFG in syntax processing more generally (e.g.(29,57–59)) by extending its role to the learning of, at least, simple scouyntactic rules. Opitz and Friederici (2004), for example, reported an increase in the activity of ventral premotor cortex following a rule violation during the learning of an artificial grammar(28). More anterior areas in the IFG have also been related to successful non-adjacent dependency learning more specifically, along with the caudate nucleus of the basal ganglia(31). Goranskaya et al. (2016), additionally, found that non-adjacent dependency learners activated a broader fronto-parietal network, including not only left frontal but also bilateral parietal regions(30). By bridging rule learning and attention, our study now sheds light onto the precise role of these regions*.”

**14. If you stimulated for 15 minutes, then the after effect should last for approx. 15 minutes after the end of the stimulation, not 30 minutes (see Siebner & Rothwell, 2003). How can you be sure that the effect was still strong enough for both tasks?**

*We thank the reviewer for pointing out this important issue. The literature regarding this topic, including the work mentioned by the reviewer (Siebner & Rothwell, 2003) but also more recent studies (Sandrini et al 2011; Valero-Cabré et al 2017), mentions that the duration of the after-effects is at least half the duration of the stimulation train (Robertson et al., 2003). This research shows that long-train low-frequency rTMS (1 Hz for 15 min) lead to neurophysiological effects that return to baseline after approximately 60% of the duration of the stimulating train itself. The after-effect can vary a lot in its duration, depending on the context (Sandrini et al 2011). The language task had a duration of 11 minutes and the attention task 12 minutes. Leading to a 23 minutes protocol, including the offline test. The same 15 min stimulation followed by 30 minutes test procedure is used in other work reporting significant effects (Rodriguez-Herreros et al 2015). In addition, we carefully limited the influence of the possible reduction of the effects at the end of the post-stimulation phase by presenting the language and attention task in counterbalanced order between participants. So, in each intervention (rTMS-POz/rTMS-lPL) half of the subjects were tested first with the attention task and half with the language task as mentioned in the manuscript p. 13, lines 260-261 and p. 30, lines 640-642 and in the caption of Figure 1, page 6 lines 101-102.*

**Reviewer #3: This paper reports an ambitious and substantial learning study using a combination of behavior, fMRI, and rTMS to try to disambiguate the cognitive/attentional processes underlying the learning of statistical regularities.  The line of research is exciting, and potentially of broad interest.
While there is a lot to recommend the paper, there are some general issues that I think should be addressed.**

*We thank the reviewer for his/her work and for the positive comments regarding our manuscript. Below, the reviewer will find point by point answers that address all his/her concerns.*

**1. -- The theoretical and empirical foundations of the 'Attention' task (using isochronous/anisochronous event timing) are much weaker than the rest of the experiments. The authors make a number of parallels between this task and non-adjacent dependencies in language (mixed rather confusingly across linguistic levels), as well as putative 'what' and 'when' pathways, but there isn't any evidence that these are related.**

*The reviewer raises two crucial points in this comment that we should had emphasized more in the previous version of the manuscript. One point is the empirical and theoretical foundations of the isochronous-anisochronous event timing as a manipulation of temporal attention. This is well-established and there is extensive research on temporal attention using this manipulation (e.g. Jones et al 2002, Sanabria et al 2011, Nobre and Van Ede 2017). We thank the reviewer for pointing out we did not sufficiently highlight this background in the manuscript. We have now referred to previous work and theoretical proposals to support it in the manuscript (page 16, lines 324-326):*

*We chose rhythm because the empirical and theoretical foundations of isochronous-anisochronous event timing are well-established as a manipulation of temporal attention[36–38].*

*The other important point that we should have emphasized is the also extensive research demonstrating that rhythm is an extremely important factor affecting language processing and language learning (p. 16, lines 326-328).*

*“In addition, there is solid evidence with respect to the effect of rhythm in speech and rule-dependency processing and learning in language, and an extensive theoretical background relating temporal attention to speech processing (e.g. Kotz, Ravignani and Fitch 2018; Kotz and Schmidt-Kassow 2015, Schon and Tillmann 2015; Selchenkova et al 2014).”*

*The contribution of our paper is precisely providing further evidence and, most importantly, putting forward a proposal that integrates language and temporal attention research in order to specify which type of temporal orienting mechanism is involved in language learning.*

*The parallels between the attention and language tasks may not have been clear enough. For attention to what: the identity of A predicts the identity of C in the language task – The identity of the first syllable of the sequence in the attention task predicts the identity of the target syllable. For attention to when (implicit): in the language task, the temporal interval between words is fully predictable such as in the isochronic condition of the attention task. Thus, in both tasks the temporal relation between elements follows the same implicit temporal expectation.*

*These definitions and the parallels between tasks have been made clearer in the manuscript in the presentation of the task (page 16, lines 317-331):*

*“In order to build a task as similar as possible to the language paradigm used for the rule learning experiment, we used syllables as the auditory stimuli on which to manipulate attention. More critically, in the language task, the temporal interval between words was fully predictable. Thus, for the Attention task, temporal orienting (when information) was manipulated by presenting the sequences of syllables in either rhythmic (with isochronous inter-stimulus intervals) or non-rhythmic form (variable time interval between syllables) while keeping the overall trial length constant. Therefore, in both tasks the temporal relation between elements follows the same implicit temporal expectation. On the other hand, in the language task, the identity of the first element informed the identity of the last element in rule blocks while this was uninformative in the no-rule blocks. Thus, in the attention task, attention to the content (what information) was manipulated by making the first syllable of the sequence informative or uninformative of the identity of the final target syllable”.*

**2. Moreover, this experiment is couched as an 'attention' manipulation but there is no independent evidence that attention is actually being directed implicitly or explicitly, which is a fairly major weakness given that the thrust of the paper is on different aspects of attention.**

*We thank the reviewer for asking for this clarification. In order to avoid this problem, we used a manipulation comparable to those previously used in temporal attention research (Sanabria et al 2011, Herbst and Obleser 2019, Cravo et al 2013). In attention experiments, goal-directed attention is explicitly manipulated by task demands: here participants were asked to focus on the identity of the first syllable since this will help them in the task. Attention can also be oriented implicitly. Participants were not informed about the rhythm manipulation. This manipulation has previously been used in studies of temporal attention research (Jones et al 2002, Sanabria et al 2011, Herbst and Obleser ,2019; Cravo et al 2013) where it has been shown that rhythm engages attention automatically and affects discrimination. Also, rhythm has repeatedly been shown to have an impact in speech processing and language learning (see response and references included in point 1 of reviewer #3). The facilitation effects based on the rhythm and identity manipulations are a direct proof of successful attention manipulation, as it is in the previous aforementioned attention studies that have similar task demands and manipulations. In all, we have emphasized these important points in the description of the task in the main text (pages 16-17, lines 332-335):*

*“Attention to the content (*what *information) was manipulated by asking the participants to focus their attention to the identity of the first syllable since this would help them to perform the task. The attention manipulations used and the orthogonality of the participant’s goal (i.e. pitch discrimination) are comparable to those previously used in temporal attention research(37,44,45).”*

*We now highlight this also in the results section (page 17-18, lines 349-354):*

*“Regarding the use of when cues, pitch discrimination in rhythmic sequences was faster than in non-rhythmic sequences (main effect of Rhythm: F(1,18) = 24.62, p < 0.001). Concerning the effect of what cues, there was also a facilitation in reaction times for informative compared to non-informative sequences (main effect of Identity: F(1,18) = 9.01, p = 0.008). The main effects of Rhythm and Identity thus indicated that the attentional manipulations were effective and participants benefited from the use of both* what *and* when *information.”*

**3. Finally, as the authors note, the results of this experiment are not particularly straightforward.  While I understand the general idea behind the experiment (and am sympathetic to it), I would recommend dropping this experiment from the paper as it - at least to my mind - detracts from the main findings, and would require a lot of extra work to properly integrate into the study.**

*Based on the previous criticisms raised by the reviewer, we fully understand this remark. We hope that the previous points, the response to point 4 of reviewer #4 (below) and response to point 2 of reviewer #2 can convince the reviewer of the relevance of the attention experiment. In our opinion, one of the major contributions of this work is that we not only show that the left parietal lobe is a key region for rule generalization, but we also unravel the underlying mechanisms that allow this generalization: the integration between what and when information. This is a major breakthrough since this mechanistic explanation is lacking in the language learning literature and allows making a direct link with attentional processes. If we remove this experiment, we are left with no direct data related to the role of the lPL we hypothesized. We can only conclude on the relation to generalization but cannot provide any explanation of the underlying mechanism sustained by this brain region in relation to temporal orienting.*

*We also think it is not ethical to remove the results of one experiment that was designed to test a specific hypothesis in the study. We cannot remove the experiment based on the fact that the results are not straightforward. Nevertheless, in an attempt to satisfy the reviewer concerns and also those raised in point 1 of Reviewer #4 while maintaining the experiment, we have toned down the conclusions throughout the manuscript and inserted a paragraph in the discussion with the limitations of this experiment for our conclusions (see also the modifications mentioned in response to point 1 Reviewer #4):*

*(p. 23, lines 477-480): “We were careful to design the attention task to match the language task as closely as possible, and the results obtained were predicted by our hypotheses. Despite this, we acknowledge that the fact that the same lPL region has both an effect on generalization and in the integration of what and when information does not necessarily imply that the two functions are linked. It could simply be due to the lPL supporting both functions without them being related. Nevertheless, the results of the attention task support our hypothesis of the lPL being relevant for the integration of* what *and* when *information, a mechanism that we claim is key in the rule generalization process.”*

*(p. 21, lines 492-497): “In conclusion, our data supports the view of two distinct stages in rule-learning, predominantly characterized by the engagement of stimulus-driven and goal-directed attention, respectively. Importantly, we have established a causal link between left parietal activation as engaged in later learning stages and the abstraction and subsequent generalization of language rules. Interfering with this region caused both an impairment in goal-directed attention and in the ability to generalize rules in language.”*

**4. On a related note, in Results, paragraph 2: "The hotspot in IPL was individually identified from the fMRI scans of the secod [typo] part of Session 1, and was assumed to reflect goal-directed attention engaged at a later stage of learning, following Part 1."   This assumption needs considerably more grounding.**

*We thank the reviewer for pointing this out (and catching the typo). We have now updated the manuscript so that it (rightly) emphasizes that we* predicted *(rather than* assumed*) that the identified area in the lPL reflects the engagement of goal-directed attention, a prediction which we later tested with the purposely designed attention task and rTMS.*

*Page 6, lines 114-117 now read:* ‘The hotspot in IPL was individually identified from the fMRI scans of the second part of Session 1. We predicted this to reflect goal-directed attention engaged at a later stage of learning, following Part 1. Targeting the lPL with rTMS in Session 2 was thus expected to...’

**5. There is quite a lot of discussion of laterality in the paper (vis-a-vis attentional networks), but no formal tests of laterality, and the rTMS was delivered only to the left. I would recommend pruning these as they do not add to the paper, and indeed confuse the issue as it makes the reader expect that hemispheric differences will be addressed when they are not.**

*As mentioned throughout the manuscript, we chose to stimulate this specific site and not others within the network of attention and the left hemisphere instead of the right hemisphere for specific theoretical reasons. This does not mean that other areas within the network in the contralateral site do not play a role. This issue is out of the scope of this work and we thank the reviewer for pointing out that we should have been more explicit about this. We have now changed the discussion in that sense (p. 22, lines 461-463):*

*“It is important to mention though that the causal role of this region does not imply that other brain areas within this network, including the contralateral parietal lobe, do not play a role. Further research is necessary to answer this question.”*

**6. The reaction time measures are (sensibly) the main evidence for online learning.  Rather surprisingly given the known problems with this approach, mean RTs are used rather than another measure.  I would recommend at least running the statistics with medians or a more sophisticated RT analysis (e.g., diffusion decision or similar).  Also, the authors might want to investigate what happens to the regression line when trials are grouped into 4 or 8 bins (for instance).**

*While RTs are, as the reviewer points out, the main evidence for online rule learning, we note that* mean *RT are solely used to measure rule effects. For the remaining analyses, we used a mixed model approach to test for online learning as trial-wise RTs gains (rather than mean RTs over blocks or bins). We refer the reviewer to the response 7 to Reviewer #2 for this matter.*

*In addition, as detailed throughout the manuscript, the regression lines are estimated from these mixed model analyses, which take into account the data for all trials, and never from binned data. Figure 3 and Figure 5 (and Figure S1 and S2) do show the data in trial bins for the sake of making the plots more intelligible (plotting all data points felt extremely cluttered). This binning was done only for visualization purposes, and this is specified now more clearly in the corresponding figure captions as follows:* ‘Actual data shown averaged into 6 trial bins (for visualization purposes only; the analysis did not bin the data) with the SEM, over the slopes for rule and no-rule derived from the mixed model analysis.’

**7. -- There is one fairly large anomalous and unaddressed RT finding, namely that the intervention group is so much faster (>100ms) in Session 2 than session 1, while the control group is not.  Assuming this is not a mistake in the x-axis of Figure 5, this needs to be tackled.**

*We thank the reviewer for raising this point. We attribute the difference between Session 1 and Session 2 for the Intervention group to non-specific rTMS effects (Duecker et al., 2013). These differences could also derive from the lower latency of the equipment employed in the rTMS facilities. However all our results are based on the differences between conditions therefore are not affected by this effect. We have now pointed out this issue in the manuscript (pp. 15, lines 299-302):*

*“Finally, we note that overall faster RTs for rTMS conditions compared to RTs for the Control group and may be due to non-specific rTMS effects(35) and/or the lower latency equipment employed for testing in the rTMS facilities do not affect the main outcome.”*

*On a side note, and thanks to the reviewer’s comment, we caught a mistake in the tick labels of the Y axis in Figure S2, which we have appropriately corrected.*

**8. The structure of the main statistical learning task is extremely simple, and on the face of it, I would have thought that most learning have happened within 15-20 seconds, and would be driven entirely by explicit strategies. Here, "Two different A_C dependencies (A1_C1 and A2_C2) were created out of 4 words from the total word pool."  Unless I have misunderstood, in a 'rule' block, the same two word pairs are repeated 48 times (with a single random word intervening between the pair). The words are bisyllabic and with native (Spanish) phonotactics and pronunciation - so learning the two word pairs should almost surely happening within the first 20 or so trials of the first block. Despite this, the offline test of this learning (as described in supplementary materials) showed very low average d-prime, which is particularly surprising after the first session where only two word pairs are learned over and over. This might be due to catastrophic interference during the test itself, but did make me wonder whether the participants were learning that the words within each pair were associated, but without attending to order. This would be easy to disambiguate by comparing responses to incorrectly mixed word pairs versus word pairs that were experienced by in the reverse order.**

*We are sympathetic to the reviewer’s perplexity at the low d’ scores achieved by participants given the apparent simplicity of the task. Two points merit emphasis in this respect. First, participants are unaware of any dependencies and their task is orthogonal to learning (word monitoring). Learning is therefore incidental. Second, the d’ scores and the corresponding % of correct responses (which are more commonly used in this literature) we obtained are comparable with previous studies employing similar designs, by our group and others (e.g. López-Barroso et al., 2016; Grama et al 2016). Nevertheless, we have now included separate d’ scores for order violations or dependency violations to answer to the reviewer concern and to point 2 of Reviewer #1 (see Supporting Information and Table S4). The results of this new analysis indicate that participants are sensitive to the order (i.e. position) of the elements in the phrase in all groups and conditions.*

**9. One general issue with the paper is that there is so much data that only some analyses get covered (I am sure to try to avoid reader overload). For instance, in fMRI study, because there are no main effects presented or graphs of the contrasts, it is not possible to know if the correlations are occurring in regions where there is activation, deactivation, or no main effect relative to baseline.   For instance, the correlations in the left IPL could be driven by relative decreases in deactivation, as opposed to activation above baseline.**

As the reviewer correctly assumes, we did not include more fMRI analyses to avoid reader overload. The correlations in the ventral fronto-parietal network (learning slopes) are driven by increases in activation above baseline during Rule blocks. We agree, however, that the work will benefit from this and we now include the following figure in the SI to clarify the point mentioned by the reviewer:

**Fig. S4. Overlap (yellow) between the ventral network correlating with Statistical Learning (green) and the main rule effect (Rule vs. Baseline; in green).** In left ventral frontal and parietal regions there is an overlap between the contrast showing regions in which the BOLD signal significantly co-varies with the measure of statistical learning (Learning Slope) and the contrast showing the brain regions in which activity increases during Rule blocks: the more activity in these regions during rule blocks, the greater (i.e. more negative) the slope. Only significant results (*p* < 0.05 FWE-corrected at the cluster level, with an additional *p* < 0.001 at the voxel level and 50 voxels of cluster extent) are shown over a canonical template with MNI coordinates on the bottom right of each slice. L, Left Hemisphere.

**Reviewer #4: In this study rule-learning and generalization was assessed in an artificial language learning task. 22 participants first trained on a first language, while their fMRI activity was recorded. In a second training session the participants learned the same rule in a second and third language, and using rTMS the role of the left parietal cortex during the generalization of this rule was assessed, as well as its role during an additional attentional task. The behavioral results of the intervention group were compared to that of a control group. The authors conclude that for rule-learning there is first a statistical learning stage, followed by a goal-directed attention stage during which the left parietal regions are involved, integrating what & when stimulus information to facilitate rule generalization.**

**This is a neat study that nicely integrates theoretical views of literature on linguistics and attention. They show that using rTMS stimulation over the parietal cortex interferes with rule generalization, giving novel insight into the neural underpinnings of rule learning. Description of methods and statistics is good.**

*We thank the reviewer for his/her work and for the positive comments regarding our manuscript. We appreciate the praise given to our attempt at integrating theoretical views of literature on linguistics and attention.*

**1. My main points for improvement concern the interpretation of the results. The authors reason/theorize that during rule-learning there is first a statistical learning stage, followed by a goal-directed attention stage. The interpretation of their results is then consistently in keeping with this terminology. However, the authors have not actually directly assessed goal-directed attention, and I am not convinced that they can label their findings as such. In doing so, they often over-interpret their results. Conclusions need to be tuned down; the results are interesting enough without over-interpretation. For example: the authors put nice predictions in Figure 2, that then hold up very nicely in the results plotted in Figure 2. On page 6 they then pose: "The combined findings of a non-significant learning slope (significantly flatter than that for Part 1) and a significant rule effect in Part 2 supports the view of a transition from statistical learning to goal-directed attention over successive rule learning parts of Session 1, as predicted.". I would agree that the results nicely show the rule-learning process: first there is statistical learning, and then the rule is known and the rule effect remains. However, in my mind there is no 'goal-directed attention' needed to explain these results. Please be careful in over-interpreting throughout the manuscript, also e.g. on page 13, and at the bottom of page 14: "we have established a causal link between goal-directed attention as engaged in later learning stages and the abstraction and posterior generalization of language rules": this is really too strong: you have established a causal link between left parietal cortex activation in later learning stages and rule generalization: this is really interesting in itself already, but you have not directly tested 'goal-directed attention', so you cannot label it as such. The parietal lobe is chosen as an rTMS stimulation site because of its involvement in temporal goal-directed attention. The resulting effects on behaviour are insightful for its role in rule-learning, but to say you are THUS showing goal-directed attention, is reverse inference. For example, the results shown in Figure 5 in the rTMS LPL group mainly show the rule is not generalized and has to be learned again, but whether this is because of less goal-directed attention, or e.g. because of the rule-being-gone or other transfer/generalization/mapping-difficulties cannot really be concluded.**

*We thank the reviewer for raising these important points. We have now updated the manuscript as follows to avoid over-interpreting our results.*

*Page 6, line 114-116: ‘The hotspot in IPL was individually identified from the fMRI scans of the second part of Session 1. We predicted this to reflect goal-directed attention engaged at a later stage of learning, following Part 1.’*

*Page 10, lines 194-195: ‘The combined findings of a non-significant learning slope (significantly flatter than that for Part 1) and a significant rule effect in Part 2 supports the view of rule-learning as a two-stage process.’*

*Page 24, lines 494-495: ‘Importantly, we have established a causal link between left parietal activation as engaged in later learning stages and the abstraction and subsequent generalization of language rules. ‘*

*However, the purpose of the attention task was in fact to be able to better specify the process interfered by the rTMS lPL intervention. Very similar tasks have been employed to explicitly assess goal-directed attention including the same manipulations and the same instructions to manipulate attention. We did not make this point clear enough. We have now clarified this (see points 1 and 2 to reviewer 3, above) to give a stronger support that we did manipulate attention and this manipulation was effective.*

*Nevertheless, we agree that the fact that rTMS interfered both with goal-directed attention as well as rule generalization does not require that the more general process (goal-directed attention) is involved in rule generalization. Because of this, we have now toned down our conclusions to read:*

*Page 13, lines 254-256: ‘(ii) whether this area was indeed involved in goal-directed attention for the binding of what and when information.’*

*Page 24, lines 495-497: “Interfering with this region caused both an impairment in goal-directed attention and in the ability to generalize rules in language.”*

*Page 23, line 471: “Finally, we devised an attention task to clarify the mechanisms underlying the lPL function”*

*We have also changed the question in the title page 13, line 253 from “Is the attention related network causally related to rule generalization to “Is left parietal lobe causally related to rule generalization and goal-directed attention?” in order to more accurately reflect what we did in our manipulation we tested whether left parietal lobe was causally related to each of these processes.*

*(see also response to point 3 to Reviewer #3)*

**2. Individual differences in the change in the rule effect from Part 1 to Part 2 were correlated with the rule vs no-rule fMRI contrast, showing a dorsal bilateral fronto-parietal network. This is then interpreted as 'consistent with the engagement of goal directed attention' (page 9). However, as you have correlated an individual difference measure here, doesn't it only mean that for SOME participants (the ones with the largest rule effect increment) there is the involvement of the dorsal bilateral fronto-parietal network? Does this hold at the group-level? Can you also present the results of the main rule effect (rule vs no-rule fMRI contrast) at the group level, to show whether a similar network was active for rule-learning in the whole group?**

*As hinted by the reviewer, since we use an individual difference measure here, this effect is greater in the participants who have already learned the rules and have a flatter slope and a greater Rule Effect. Given this variability in learning, the only way to assess brain activity is to use an individual differences approach. Studying this group of participants as a group knowing that they differ behaviorally in the measure that is related to goal-directed attention would not be appropriate: the participants’ learning behavior is too different. Indeed, the dorsal network shown only holds at the group level at uncorrected levels, something expected given that some participants have already learned the rule dependencies (larger Rule Effect) and others still have significant learning slopes (related to statistical learning). Knowing from all the previous work on language learning that these individual differences were expected that is also the reason we used the approach to select the peak of activation at the individual level for the rTMS stimulation.*

**3. With the use of the attention task the authors nicely try to interpret the role of the left parietal lobe during rule learning by looking at its role during another task. As far as I understood from the methods section, the control group also did this attention task. What were their results? Were they comparable to the POz rTMS results, or did the POz rTMS result in again another pattern?**

*We appreciate the reviewer’s understanding of our approach. Unfortunately, the Control group did not perform the attention task. As mentioned above in response to point 1 to Reviewer 2,* *the Control group was included to assess task repetition effects, and control for the undesired effects that performing the task in the MRI scanner or in a TMS setting could have on learning; that is, to provide a baseline for the effect of rule generalization without any form of intervention (fMRI or rTMS). The performance of this group was critical to evaluate the potential effects of rTMS POz, and to legitimate the suitability of this condition (rTMS POz) as a control rTMS condition in the learning task. We have now made this clearer in the main body of the manuscript as well as in the methods section (Please refer to the response to point 1 of Reviewer 2 above).*

**4. Although I do really appreciate the use of the attention task to pin down the exact function of this area, again, be careful with reverse inference: if two tasks are distorted because of rTMS to a particular site, this does not necessarily mean that the two tasks are similar.**

*We fully understand the reviewer’s concern, and this is an aspect we were aware of when designing this project. In order to try to limit as much as possible this problem, we tried to make the attention task as similar as possible to the language task but following the design and task of previously used temporal attention tasks. Although the task in the attention experiment was different (pitch discrimination task as opposed to a target discrimination), we maintained a close parallel between the two tasks. Namely, we used linguistic material in both tasks (syllables vs words). The focus of attention was on identity of the syllables in the attention task while the focus on the identity of the words was necessary to be able to learn. The presentation of the syllables followed an isochronic relation as in the language task. The stimulation site was chosen at the individual level from the same subjects performing the language learning task in the fMRI. The attention task was performed by the same subjects and under the same rTMS intervention (i.e. the language and attention tasks were performed under the after-effects of the same rTMS offline intervention). In addition, we had a precise hypothesis when designing the attention task with these precise characteristics. Despite all that, it is certainly true that it is not exactly the same task, and these effects, even when predicted by our hypothesis, could simply be due to the same area supporting different unrelated functions. We have accordingly toned down by rephrasing our conclusions in this respect and we have included a discussion of this limitation in the discussion section (page 23, lines 477-484):*

*“We were careful to design the attention task to match the language task as closely as possible, and the results obtained support our hypothesis. The lPL was proven to be relevant for the integration of what and when information, a mechanism that we claim is key in the rule generalization process. The exact same region was critical for rule generalization. Despite this, we acknowledge that the fact that the same lPL region has both an effect on generalization and in the integration of what and when information does not necessarily imply that the two functions are linked. It could simply be due to the lPL supporting both functions without them being related. Further investigations will shed light onto this issue.”*

Other changes:

1. In addition to the references that follow at the end of this letter we added a recent reference briefly commented now in the discussion *(Summerfield C, Luyckx F, Sheahan H. Structure learning and the posterior parietal cortex. Progress in Neurobiology. Elsevier Ltd; 2020. p. 101717. doi:10.1016/j.pneurobio.2019.101717)*. This proposal, based on deep learning networks and vision research, argues for a very similar but more general role of the parietal cortex in the encoding of the relative position of objects or features in the physical space and the relations among entities in the abstract conceptual space.

***References***

*Saffran JR, Wilson DP. From syllables to syntax: Multilevel statistical learning by 12-month-old infants. Infancy. 2003;4(2):273–84.*

*Saffran, J. R. (2003). Statistical Language Learning: Mechanisms and Constraints. Current Directions in Psychological Science, 12(4), 110–114.* [*https://doi.org/10.1111/1467-8721.01243*](https://doi.org/10.1111/1467-8721.01243)

*Richter D, De Lange FP. Statistical learning attenuates visual activity only for attended stimuli. 2019 [cited 2020 Apr 18]; Available from:* [*https://doi.org/10.7554/eLife.47869.001*](https://doi.org/10.7554/eLife.47869.001)

*van Moorselaar D, Slagter HA. Learning What Is Irrelevant or Relevant: Expectations Facilitate Distractor Inhibition and Target Facilitation through Distinct Neural Mechanisms. J Neurosci. 2019 Aug 28;39(35):6953–67.*

*Toro JM, Sinnett S, Soto-Faraco S. Speech segmentation by statistical learning depends on attention. Cognition. 2005;97(2):25–34.*

*Lieberman,M.D. & Cunningham,W.A. Type I and Type II error concerns in fMRI research: re-balancing the scale. Soc. Cogn Affect. Neurosci. 4, 423-428 (2009).*

*Duecker F, de Graaf TA, Jacobs C, Sack AT (2013) Time- and Task-Dependent Non-Neural Effects of Real and Sham TMS. PLoS ONE 8(9): e73813.* [*https://doi.org/10.1371/journal.pone.0073813*](https://doi.org/10.1371/journal.pone.0073813)

*Karuza, E. A., Emberson, L. L., & Aslin, R. N. (2014). Combining fMRI and behavioral measures to examine the process of human learning. Neurobiology of learning and memory, 109, 193–206. https://doi.org/10.1016/j.nlm.2013.09.012*

*Siegelman N, Bogaerts L, Kronenfeld O, Frost R. Redefining "Learning" in Statistical Learning: What Does an Online Measure Reveal About the Assimilation of Visual Regularities? Cognitive Science. 2018 Jun;42 Suppl 3:692-727. DOI: 10.1111/cogs.12556.*

*López-Barroso D, Cucurell D, Rodríguez-Fornells A, de Diego-Balaguer R. Attentional effects on rule extraction and consolidation from speech. Cognition. 2016;152:61–9.*

*Grama, I, Kerkhoff, Wijnen. Gleaning Structure from Sound: The Role of Prosodic Contrast in Learning Non-adjacent Dependencies. (2016). J Psycholinguist Res. DOI 10.1007/s10936-016-9412-8*

*Harris IM, Benito CT, Ruzzoli M, Miniussi C. Effects of right parietal transcranial magnetic stimulation on object identification and orientation judgments. J Cogn Neurosci [Internet]. 2008 May [cited 2020 Apr 18];20(5):916–26. Available from:* [*http://www.ncbi.nlm.nih.gov/pubmed/18201128*](http://www.ncbi.nlm.nih.gov/pubmed/18201128)

*Ruzzoli M, Soto-Faraco S. Alpha stimulation of the human parietal cortex attunes tactile perception to external space. Curr Biol [Internet]. 2014 Feb 3 [cited 2020 Apr 18];24(3):329–32. Available from:* [*http://www.ncbi.nlm.nih.gov/pubmed/24440394*](http://www.ncbi.nlm.nih.gov/pubmed/24440394)

*Marigold DS, Lajoie K, Heed T. No effect of triple-pulse TMS medial to intraparietal sulcus on online correction for target perturbations during goal-directed hand and foot reaches. PLoS One [Internet]. 2019 [cited 2020 Apr 18];14(10):e0223986. Available from:* [*http://www.ncbi.nlm.nih.gov/pubmed/31626636*](http://www.ncbi.nlm.nih.gov/pubmed/31626636)

*Capotosto P, Corbetta M, Romani GL, Babiloni C. Electrophysiological correlates of stimulus-driven reorienting deficits after interference with right parietal cortex during a spatial attention task: a TMS-EEG study. J Cogn Neurosci [Internet]. 2012 Dec [cited 2020 Apr 18];24(12):2363–71. Available from:* [*http://www.ncbi.nlm.nih.gov/pubmed/22905824*](http://www.ncbi.nlm.nih.gov/pubmed/22905824)

*Valero-Cabré A, Amengual JL, Stengel C, Pascual-Leone A, Coubard OA. Transcranial magnetic stimulation in basic and clinical neuroscience: A comprehensive review of fundamental principles and novel insights. Neurosci Biobehav Rev [Internet]. 2017;83(July):381–404. Available from:* [*https://doi.org/10.1016/j.neubiorev.2017.10.006*](https://doi.org/10.1016/j.neubiorev.2017.10.006)

*Sandrini et al. The use of transcranial magnetic stimulation in cognitive neuroscience: A new synthesis of methodological issues. Neuroscience and Biobehavioral Reviews 35, 516–536 (2011).*

*Siebner & Rothwell. Transcranial magnetic stimulation: New insights into representational cortical plasticity. Experimental Brain Research 148, 1–16 (2003).*

*Robertson et al. Studies in Cognition: The Problems Solved and Created by Transcranial Magnetic Stimulation. J. Cogn. Neurosci. 15, 948–960 (2003).*

*Rodríguez-Herreros et al. Microstructure of the superior longitudinal fasciculus predicts stimulation-induced interference with on-line motor control. Neuroimage 120, 254–265 (2015).*

*Jones MR, Moynihan H, MacKenzie N, Puente J. Temporal Aspects of Stimulus-Driven Attending in Dynamic Arrays. Psychol Sci [Internet]. 2002 Jul 6 [cited 2020 Apr 18];13(4):313–9. Available from:* [*http://journals.sagepub.com/doi/10.1111/1467-9280.00458*](http://journals.sagepub.com/doi/10.1111/1467-9280.00458)

*Sanabria D, Capizzi M, Correa Á. Rhythms That Speed You Up. J Exp Psychol Hum Percept Perform [Internet]. 2011 Feb [cited 2020 Apr 18];37(1):236–44. Available from:* [*http://www.ncbi.nlm.nih.gov/pubmed/20718571*](http://www.ncbi.nlm.nih.gov/pubmed/20718571)

*Nobre AC, Van Ede F. Anticipated moments: Temporal structure in attention [Internet]. Vol. 19, Nature Reviews Neuroscience. Nature Publishing Group; 2018 [cited 2020 Apr 18]. p. 34–48. Available from:* [*http://www.ncbi.nlm.nih.gov/pubmed/29213134*](http://www.ncbi.nlm.nih.gov/pubmed/29213134)

*Kotz SA, Ravignani A, Fitch WT. The Evolution of Rhythm Processing [Internet]. Vol. 22, Trends in Cognitive Sciences. Elsevier Ltd; 2018 [cited 2020 Apr 18]. p. 896–910. Available from:* [*http://www.ncbi.nlm.nih.gov/pubmed/30266149*](http://www.ncbi.nlm.nih.gov/pubmed/30266149)

*Kotz SA, Schmidt-Kassow M. Basal ganglia contribution to rule expectancy and temporal predictability in speech. Cortex. 2015 Jul 1;68:48–60.*

*Selchenkova T, François C, Schön D, Corneyllie A, Perrin F, Tillmann B. Metrical Presentation Boosts Implicit Learning of Artificial Grammar. Troyer TW, editor. PLoS One [Internet]. 2014 Nov 5 [cited 2020 Apr 18];9(11):e112233. Available from:* [*http://dx.plos.org/10.1371/journal.pone.0112233*](http://dx.plos.org/10.1371/journal.pone.0112233)

*Schön D, Tillmann B. Short- and long-term rhythmic interventions: Perspectives for language rehabilitation. Ann N Y Acad Sci [Internet]. 2015 Mar 1 [cited 2020 Apr 18];1337(1):32–9. Available from:* [*http://doi.wiley.com/10.1111/nyas.12635*](http://doi.wiley.com/10.1111/nyas.12635)

*Herbst SK, Obleser J. Implicit temporal predictability enhances pitch discrimination sensitivity and biases the phase of delta oscillations in auditory cortex. Neuroimage [Internet]. 2019 Dec 1 [cited 2020 Apr 18];203:116198. Available from:* [*http://www.ncbi.nlm.nih.gov/pubmed/31539590*](http://www.ncbi.nlm.nih.gov/pubmed/31539590)

*Cravo AM, Rohenkohl G, Wyart V, Nobre AC. Temporal expectation enhances contrast sensitivity by phase entrainment of low-frequency oscillations in visual cortex. J Neurosci. 2013 Feb 27;33(9):4002–10*
