# Supplementary material for: Integrating when and what information in the left parietal lobe allows language rule generalization
Source: PLoS Biol. 2020 Nov 2;18(11):e3000895. doi: 10.1371/journal.pbio.3000895 (PMC7660506; doi:10.1371/journal.pbio.3000895)
Supplement: Attachment — Submitted filename: Response_to_Reviewers_2.docx [file pbio.3000895.s019.docx]

Reviewer remarks:

**Reviewer #1**, **Jutta Mueller**:

1. **Regarding my concerns with respect to interpretation of the experimentally induced learning processes as statistical learning the authors responded with a rebuttal. Yet, I am not convinced by the response and would like to continue the debate (especially if we would sit together in person). I am a bit drawn between disagreeing with how the term statistical learning is used and the simultaneous impression that this is not a major issue affecting the really good quality and insightfulness of the present study. As the authors cite Jenny Saffran themselves "statistical learning capitalizes on the statistical properties of the language environments". Yet, if pauses structure the input stream it is not only statistical properties of the language environment that is used to capitalize on but also additional perceptual properties. Of course one could argue now that perceptual properties also have a statistical distribution, but then the original idea of statistical learning is somehow distorted, namely that the distributional properties of the information to be learned suffices for learning and not, as I understand it, the distributional properties of the cues to the information to be learned.**

*We thank the reviewer for the interesting debate, and we look forward for in-person discussions that will with no doubt enrich our views on the topic. With respect to this first issue, we see many points of agreement with the reviewer, but we may not have been clear enough in relation to how perceptual cues relate to statistical learning. As the reviewer states, perceptual cues structure the input, but we believe this has different effects depending on where these pauses occur. The comment of the reviewer has been very insightful since we believe an explanation in terms of stimulus-driven/goal-directed attention may be helpful to clarify our view. On the one hand, pauses have a stimulus-driven effect: when no pauses are present, the speech stream is perceived as a sequence of syllables; when pauses occur between words, the speech stream is perceived as a sequence of words. However, this does not mean that the SL mechanism per se changes; it changes the units to which the SL is applied. In other words, the same SL mechanism that detects co-occurrences can be applied to either syllables or words. As the reviewer later comments, our view is that SL is essentially a stimulus-driven process, and as such relates to stimulus-driven attention, in the way that this type of pauses that perceptually segment the speech stream drive perceptual effects.*

*On the other hand, it is also true that pauses can also be used as cues to attract attention to nearby elements when they structure the input in particular ways. In the specific case of our material, pauses longer than those between words group sequences of words into phrase units, and they happen to occur at utterance edges where dependencies lie. These type of pauses that concur with relevant information (e.g. Peña et al., 2002), as much as other types of cue that have been reported in the literature (e.g. changes in pitch: Creel et al., 2004; high X variability: Gómez, 2002; Onnis et al., 2004) are essential for rule abstraction and generalization (Peña et al., 2002; Endress and Bonatti, 2006; Onnis et al., 2004). Our view is that these cues help orient (goal-directed) attention towards the relevant (dependency-bearing) stimuli for a computational stage subsequent to SL (i.e., rule abstraction for generalization). Our goal here was to show both that this attentional deployment occurs and is critical.*

*Concerning “Statistical Learning”, it is unknown whether SL is an actual ‘mechanism’ in itself or, instead, an observation of learning that reflects the structure of the input. Note that the latter case implies a more general learning mechanism that encompasses the learning of statistical regularities (cf. Schapiro et al. 2013). What the mechanism of SL is, in itself or as part of a more general learning system, is not the focus of the paper. Instead, we focus on describing two distinct stages that are involved in language rule learning and generalization. In this respect, our claim with regards to SL is limited to stating that the first of these stages reflects the (stimulus-driven) learning of the statistical relations between the presented stimuli.*

*In all, we believe that both point 1 and 2 of the reviewer (see below for the response to point 2) can be addressed in a simpler way by following her suggestion to further highlight the distinction between an early* ***stimulus-driven*** *stage linked to SL and a later goal-directed attention stage.*

2. **The other problem that I have as a reader relates to the idea of a shift from a learning mechanisms, statistical learning (of which I don't think that it only includes learning of TPs), to goal-directed attention, which can be an ingredient to many different cognitive processes. The two processes, statistical learning and goal-directed attention, are in my view not on the same granularity level. I would conceptualize the present study rather generally as capturing a transition from call-it stimulus-driven learning to attention-driven processing and generalization. Somehow I have the impression that this is not so far from what the authors assume. In the conclusion the authors write about the engagement of stimulus-driven and goal-directed attention - with this it is much easier to agree. Here are several solutions to the problem: 1) The authors could define at the beginning how exactly they use the terms statistical learning and goal-directed attention and/or 2) The authors could just stick with their very consistent interpretation of a two-staged process from learning, which might be more stimulus-driven, to processing and generalization, which might be more driven by endogenous attention. Solution # 3 would be that I accept the authors' interpretation as an instance of a naturally occurring disagreement in science that does not affect the methodological quality of the presented experiment, which would be also fine.**

*In order to answer this and the previous point of the reviewer, we have emphasized throughout the introduction the distinction between stimulus-driven SL and goal-directed attention and how these two different mechanisms (and networks) are engaged in the early stages of learning and in the later stages of learning and generalization, respectively. As suggested by the reviewer, therefore, we have established a clearer distinction between statistical learning as a stimulus-driven process and a goal-directed attention that engages later in the learning process (line 5 in the abstract; lines 35-36, 42-43, 51-52, 62, 67-68, 79, 84).*

**Reviewer #3**: **The authors responded copiously in the rebuttal letter to the reviewer comments, and made a number of changes to the manuscript to address points I had raised in the last review, including the theoretical background for the isochronous/anisochronous sequence task.**

1. **As with R4 in her/his last review, I still do not see a convincing argument that the dependent measure in the 'attention' task necessarily measures what typically is referred to as goal-directed attention. My own view is this would require a fair amount of behavioral work to demonstrate, so not really within the scope of the current study. However, as the authors point out, this type of manipulation has been in the literature on temporal attention. I think readers can make their own decisions on this point**.

*We agree with the reviewer that this is an intense area of debate in the literature. Although the pitch discrimination task and the instructions to the subjects to focus on the identity of the syllable can be more easily assimilated to goal-directed attention, the isochronous/anisochronous manipulation is viewed, even within the temporal attention literature, as having both an exogenous and an endogenous component (Nobre and van Ede, 2018). We did not want to enter this debate here. Our aim was to use a manipulation of attention that was as close as possible to the language task and to manipulate goal-directed attention as much as possible combining what and when features. We were careful to not describe the isochronous/anisochronous manipulation specifically as goal-directed attention. We agree with the reviewer that the best is to refer the reader for details to the temporal attention literature. We thank again the reviewer for the insightful comments.*

2. **Comment and question: Lines 196-198. "The combined findings of a non-significant learning slope (significantly flatter than that for Part 1) and a significant rule effect in Part 2 supports the view of rule-learning as a two stage-process." How is this a 'two-stage process' of learning, as opposed to 'learning' and sustained retention of that learning? Or indeed, just an ongoing process of learning, given this statement: "An analysis of the Intervention and Control groups separately indicated a similar pattern of results in the two groups, except that in the Intervention group some learning was still in progress in Part 2".**

*The interpretation of the reviewer is fully compatible with our view of a two-stage process. The idea of a statistical learning mechanism that extracts the co-occurrences in the input allows, at a later stage, to focus on the elements that have been learned to predict the forthcoming element of the dependency. In the specific case of the artificial language used, in the second stage (what the reviewer sees as a “sustained retention of the learning”) learners have already extracted the relations and can apply their knowledge by simply focusing their attention on the first and last elements of the phrase. We thank the reviewer for pointing out the statement that was misleading. What we meant to convey is that some statistical learning was still going on in the intervention group and that therefore some participants were still in stage 1. We now rephrased this as (lines 201-203): “An analysis of the Intervention and Control groups separately indicated a similar pattern of results in the two groups, except that in the Intervention group some SL was still in progress in Part 2”.*

3. **With the new figure showing the fMRI activation related to rule versus non-rule learning (S3 - thanks for creating that), it is unclear how the fMRI results guided the ROI used for the TMS given that the differences in activation were basically found across a broad network of areas. Also, what was the pattern of activation near the coordinates for the POz control site?**

*As we mentioned in the introduction, we expected to find activity over a broad fronto-parietal network during the latter part of learning when participants were scanned. The decision to stimulate specifically within the left parietal lobe (lPL) was theoretically driven as we review in the introduction, given the implication of lPL in temporal attention (pages 4, last paragraph - page 5, first paragraph). However, we thank the reviewer for this comment that allowed us to notice we did not highlight clearly enough the rationale for the selection of this specific area within the fronto-parietal network. We have now introduced a sentence to clearly state why we selected this region (lines 121-123, page 6-7): “The lPL area targeted in the rTMS intervention was chosen a priori for its known role in temporal attention (Nobre and van Ede, 2018; Coull et al. 2016), which was hypothesized to play a crucial role in the generalization of linguistic rules.”*

*Concerning* *the activation of POz, Figure S3 shows the overlap of individual masks for each participant's activation pattern. As can be observed, there is no systematic pattern of activation at the POz location, neither any significant cluster at this location in the regression analyses. This important information has now been introduced in lines 265-267: “No systematic activation at the POz location was observed in the individual contrasts (see S3 Fig) or in the regression analyses (see Fig 4, S1 Table and S2 Table) confirming the appropriateness of this location as a control site.”*

*In all, we thank all reviewers for their extremely helpful work and for making this a stronger article.*

***References***

*Peña M, Bonatti LLL, Nespor M, Mehler J. Signal-driven computations in speech processing. Science (80-). 2002;298: 604–7. doi:10.1126/science.1072901*

*Gómez R. Variability and detection of invariant structure. Psychol Sci. 2002;13: 431–436. doi:10.1111/1467-9280.00476*

*Coull JT, Cotti J, Vidal F. Differential roles for parietal and frontal cortices in fixed versus evolving temporal expectations: Dissociating prior from posterior temporal probabilities with fMRI. Neuroimage. 2016;141: 40–51. doi:10.1016/j.neuroimage.2016.07.036*

*Creel, S. C., Newport, E. L., & Aslin, R. N. Distant melodies: Statistical learning of nonadjacent dependencies in tone sequences. Journal of Experimental Psychology: Learning Memory and Cognition, 2004; 30(5), 1119–1130.* [*https://doi.org/10.1037/0278-7393.30.5.1119*](https://doi.org/10.1037/0278-7393.30.5.1119)

*Onnis, L., Monaghan, P., Christiansen, M. H., & Chater, N. (2004). Variability is the spice of learning, and a crucial ingredient for detecting and generalizing in nonadjacent dependencies. Proceedings of the 26th Annual Conference of the Cognitive Science Society, (January), 1047–1052.*

*Endress, A. D., & Bonatti, L. L. (2006). Rapid learning of syllable classes from a perceptually continuous speech stream. Cognition, 105(2), 247–299.* [*https://doi.org/10.1016/j.cognition.2006.09.010*](https://doi.org/10.1016/j.cognition.2006.09.010)

*Schapiro, A.C., et al., Neural representations of events arise from temporal community structure. Nature Neuroscience, 2013.* ***16****(4): p. 486-492.*

*Nobre, A., van Ede, F. Anticipated moments: temporal structure in attention. Nat Rev Neurosci* ***19,*** *34–48 (2018). https://doi.org/10.1038/nrn.2017.141*
